# Integrated monitoring and modeling to disentangle the complex spatio-temporal dynamics of urbanized streams under drought stress

**DOI:** 10.1007/s10661-024-12666-3

**Published:** 2024-05-20

**Authors:** Gregorio Alejandro López Moreira Mazacotte, Doerthe Tetzlaff, Christian Marx, Maria Magdalena Warter, Songjun Wu, Aaron Andrew Smith, Chris Soulsby

**Affiliations:** 1 Department of Ecohydrology and Biogeochemistry, Leibniz Institute of Freshwater Ecology and Inland Fisheries (IGB), Berlin, Germany; 2 Geography Institute, Humboldt University of Berlin, Berlin, Germany; 3 MOSAIC Research Group - Modelling Surface and Groundwater With Isotopes in Urban Catchments, Technische Universität Berlin, Berlin, Germany; 4 School of Geosciences, Northern Rivers Institute, University of Aberdeen, Aberdeen, Scotland, UK; 5 IRI THESys, Humboldt University of Berlin, Berlin, Germany

**Keywords:** Urban hydrology, Stable isotopes, Runoff processes, Urban groundwater, Urban drainage

## Abstract

We have a poor understanding of how urban drainage and other engineered components interact with more natural hydrological processes in green and blue spaces to generate stream flow. This limits the scientific evidence base for predicting and mitigating the effects of future development of the built environment and climate change on urban water resources and their ecosystem services. Here, we synthesize > 20 years of environmental monitoring data to better understand the hydrological function of the 109-km^2^ Wuhle catchment, an important tributary of the river Spree in Berlin, Germany. More than half (56%) of the catchment is urbanized, leading to substantial flow path alterations. Young water from storm runoff and rapid subsurface flow provided around 20% of stream flow. However, most of it was generated by older groundwater (several years old), mainly recharged through the rural headwaters and non-urban green spaces. Recent drought years since 2018 showed that this base flow component has reduced in response to decreased recharge, causing deterioration in water quality and sections of the stream network to dry out. Attempts to integrate the understanding of engineered and natural processes in a traditional rainfall-runoff model were only partly successful due to uncertainties over the catchment area, effects of sustainable urban drainage, adjacent groundwater pumping, and limited conceptualization of groundwater storage dynamics. The study highlights the need for more extensive and coordinated monitoring and data collection in complex urban catchments and the use of these data in more advanced models of urban hydrology to enhance management.

**Supplementary Information:**

The online version contains supplementary material available at 10.1007/s10661-024-12666-3.

## Introduction

Despite being heavily managed, our understanding of the hydrological function of most urban streams is surprisingly poor (Ehleringer et al., 2016). Environmental data collection in urban areas is often limited and poorly coordinated between multiple agencies (Marx et al., 2023; Niemczynowicz, 1999). Consequently, while modeling urban storm drainage to help manage flood risk is commonplace, the complex interactions between engineered urban water management systems and what remains of the more natural hydrology of urban greenspaces and any rural hinterland have only recently been the focus of attention (Marx et al., 2022; Oswald et al., 2023). Moreover, the cumulative effects of ongoing urban (re-)development and the *memory* effects of historic management changes may be unknown (Marlow et al., 2013). This lack of scientific understanding poses challenges for the future management of urban water resources. A firm knowledge of urban hydrological function is a prerequisite to anticipating and sustainably managing emerging pressures from the population growth, urban expansion, and climate change impacts expected in many urban centers (Miller & Hutchins, 2017; Niemczynowicz, 1999).

Fortunately, the last few decades have seen increasingly integrated approaches to *catchment science*. These provide a fuller, more nuanced approach to understanding hydrological function, by integrating diverse complementary field data on the water cycle beyond simply rainfall and runoff (Hutchins et al., 2017). These include hydroclimatic fluxes (Vulova et al., 2023), isotopic and water quality characteristics (Kuhlemann et al., 2020), soil moisture and groundwater processes (Duethmann et al., 2022; Schaffitel et al., 2020), and vegetation parameters (Bernard et al., 2022; Crampe et al., 2021); data which are either empirically derived or remotely sensed at various spatial and temporal scales. Such data can then drive exploratory, multi-calibrated modeling to develop hypotheses of hydrological functions (e.g., Birkel et al., 2014; Kuppel et al., 2018). Rainfall-runoff models can then be tested against more focused field data collection and/or support more advanced modeling that goes beyond these more traditional approaches to understanding the rainfall-runoff transformation that is the basis of urban flood modeling (Green et al., 2021; Jefferson et al., 2017).

Consequently, more hydrological studies are moving towards integrating a more process-based understanding of the spatio-temporal dynamics of the urban water cycle components across the urban critical zone (Arden et al., 2014; Cristiano et al., 2020; Gillefalk et al., 2022; Salvadore et al., 2015). Studies investigating urban catchment hydrology have shown significantly lower water ages (Mean Transit Times – MTTs) and higher fractions of young water than non-urban catchment as a result of artificial storm drainage and reduced recharge and groundwater storage under impervious surfaces (Bonneau et al., 2018; Kuhlemann et al., 2022; Parajulee et al., 2019; Soulsby et al., 2015; Stevenson et al., 2022). Those impacts are likely to be time-variant as urban areas evolve (Birkel et al., 2012; Rodriguez et al., 2020). Such changes in urban flow paths and landscape connectivity can also impact water quality beyond storm runoff (Knapp et al., 2022; Zhi & Li, 2020) and are also linked to hydroclimate and urbanization (Ferreira et al., 2021; Kuhlemann et al., 2022; Marx et al., 2023). Potential effects may include passive transport of pollutants along natural and anthropogenic flow paths, impacts of urban wastewater effluents, and incidents where storm drains are connected to foul sewers which can mix and spill in combined sewer overflows during rainfall events (Oswald et al., 2023; Paton & Haacke, 2021).

Ecohydrological processes in urban green spaces have been investigated across scales, ranging from the street scale (Meili et al., 2020), to plot and sub-district level (Gillefalk et al., 2021, 2022) for addressing soil water storage dynamics and evapotranspiration, and integrating specific urban effects on ecohydrological fluxes (Meili et al., 2020; Ring et al., 2023). Urban impacts on groundwater recharge have also been investigated. For example, Oiro et al., (2018, 2020) quantified the temporal controls of the aquifer recharge and depletion of large-scale groundwater resources under the heavily depleted Nairobi aquifer system in Kenya. Other recent studies have aimed to quantify changes in subsurface water storage (Bhaskar et al., 2015, 2016), how groundwater recharge and stream flow generation processes influence urban water quality (Welty et al., 2023), and street tree water uptake (Revelli & Porporato, 2018). There remain significant challenges to integrating urban water balance components, changes in subsurface water storage, and stream flow generation processes into a “unified” urban model to address all potential hydrological processes (Oswald et al., 2023; Salvadore et al., 2015). Importantly, this needs to be done in such a way that is cognizant of historical management legacies that may continue to impact urban water.

In this study, our overall objective was to apply such an integrated approach to improve our understanding of a major urban water resource in a large city. For this, we selected the Wuhle catchment in eastern Berlin, Germany. The catchment drains an area of some 109 km^2^ and is 56% urbanized, mostly focused in its lower reaches. It has a major role in connecting urban green spaces, allotments, and amenities used in one of the less affluent parts of the city. A long and ongoing history of water management continues to affect its current function. In the context of Berlin’s urban streams, it is unique in that its flow regime does not currently receive urban wastewater from either sewage treatment plants or sewer overflows (Kuhlemann et al., 2020; Möller & Burgschweiger, 2008). The sewer and rainwater systems are generally separated, though some historic misconnections are likely (Geoportal Berlin / [Sewer System], 2017, https://fbinter.stadt-berlin.de/fb/index.jsp). Preliminary work showed that the dominant stream flow generation processes reflect the interplay of urban storm drainage and local groundwater systems recharged through urban and peri-urban green spaces (Kuhlemann et al., 2022). However, the stream is also affected by local groundwater abstractions by the local water company, the *Berliner Wasserbetriebe* (BWB), and the more recent development of a network of urban flood control storage ponds.

The specific research aims were to:

1. Characterize the hydrology of the Wuhle catchment in terms of the interactions between hydroclimate, local groundwater systems, local water management systems, and the rainfall-runoff response.
2. Use hydrological tracers, including stable water isotopes and a range of water quality parameters to assess the spatio-temporal variations in dominant water sources and flow paths.
3. Use exploratory modeling in a learning framework to integrate these insights and understand what is known about the catchment’s response to a recent drought period, particularly concerning low flows.

The study also highlights some generic issues of the need for, and challenges to, future field data collection and modeling in urban catchments to provide evidence for future management in the face of climate change and increased urban growth.

## Materials and methods

### Study catchment

**Fig. 1:**
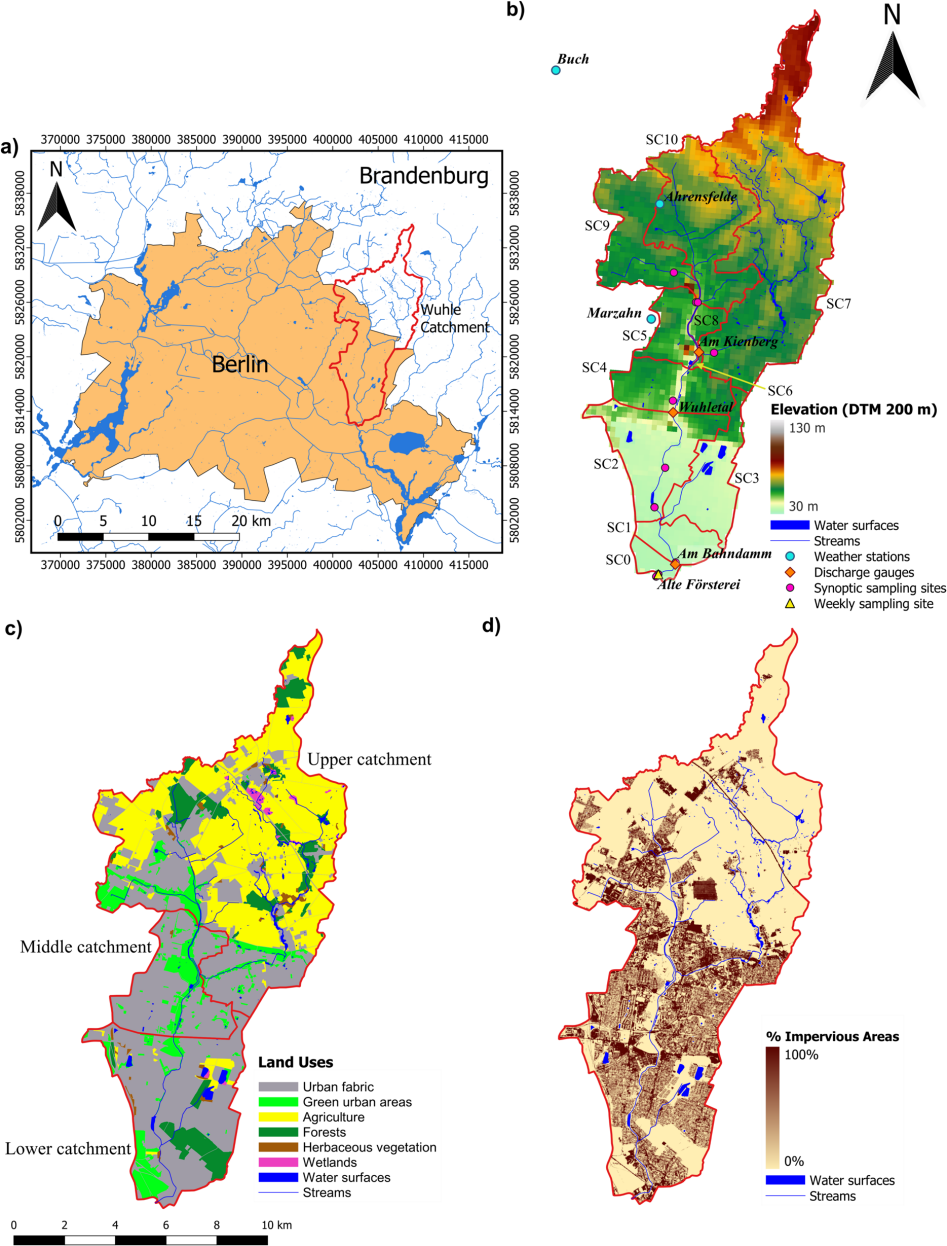
Maps showing: **a** catchment location in the Berlin-Brandenburg area in Germany, **b** elevation, monitoring and sampling sites, and modeled sub-catchments, **c** land use, and **d** percentage of the catchment with impervious cover

**Fig. 2:**
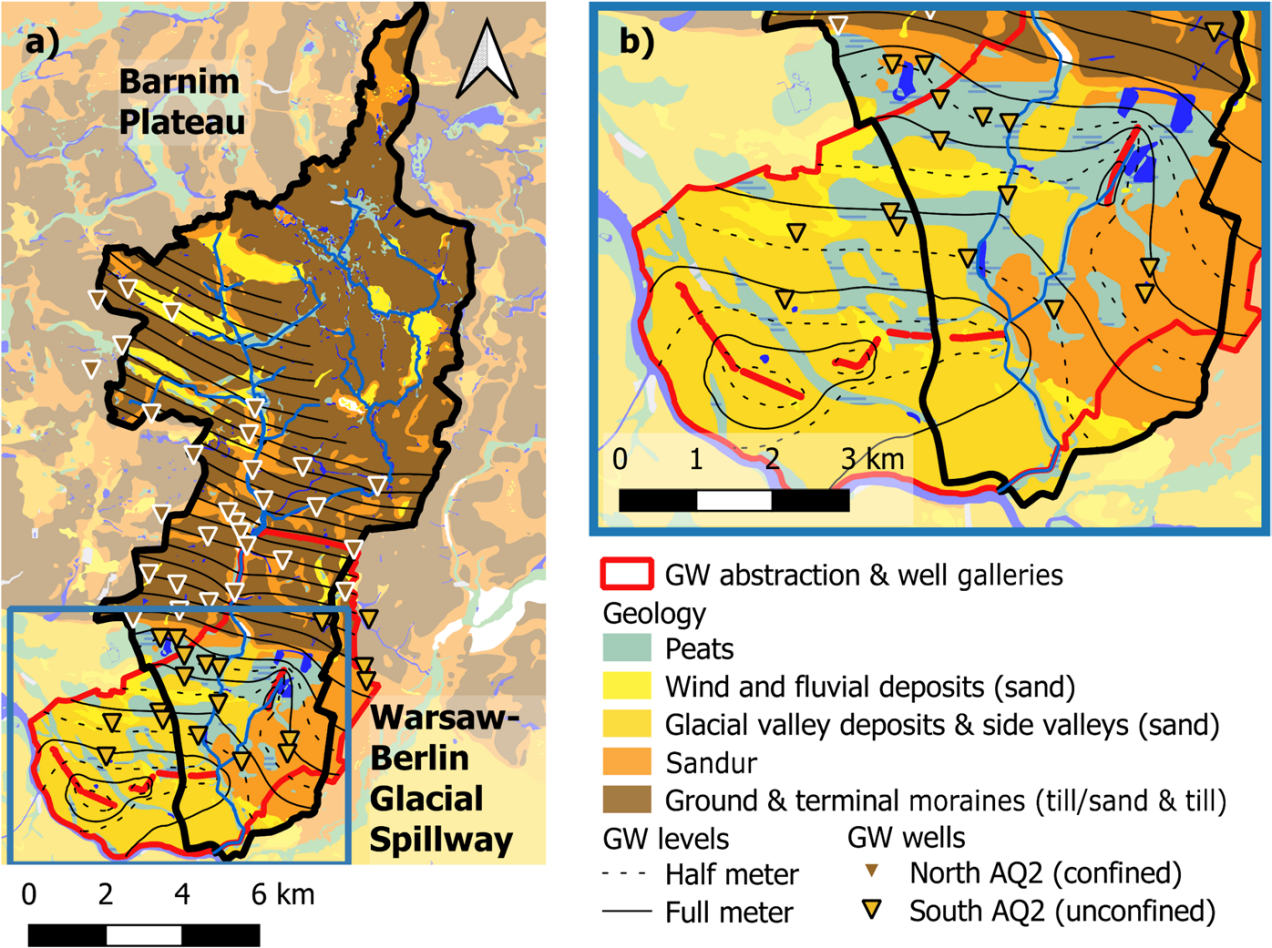
**a** Distribution of groundwater wells and water table elevations in the Wuhle catchment and **b** zoomed in on the lower catchment

The Wuhle catchment is located in Germany, in the Berlin-Brandenburg area, spanning an area of ~ 109 km^2^ (Fig. 1a). Its main channel network forms a small right-bank tributary of the river Spree, draining parts of the Barnim Plateau in the NE of Berlin (Fig. 2a). Its headwaters include natural topographic highs reaching 115 m and the Biesdorfer Heights, which are artificially elevated areas created by rubble removed from central Berlin after the Second World War.

The lower catchment forms part of the Warsaw-Berlin *Urstromtal*, a broad glacial valley (Fig. 2b). This originated during the Weichselian glaciation which ended around 11,700 years ago (Litt et al., 2007). As the ice sheet melted, water flowed south, shaping the *Wuhletal* (the Wuhle Valley), with a distinctive wide, gently sloping landscape. The upper reaches of the Wuhle Valley are located on top of the Barnim Plateau and are covered by ~ 40 m of silty, low permeability moraine, causing the underlying gravel aquifer to be confined (known as the AQ2 in Berlin) (Limberg & Thierbach, 2002). Major tributaries of the Wuhle drain these headwaters. In the lower catchment, the gravel aquifer (known as AQ1.3 + 2) is unconfined, and the prevailing hydraulic gradient results in increased groundwater discharge to the stream in this area (Fig. 2). The soils include glaciofluvial and windblown sands in the lower catchment and silty clay soils on glacial till in the upper catchment as well as areas of peat and lacustrine soils which form part of the corridors of urban green space fringing the river channel in the Wuhle Green Belt (Fig. 1c).

**Fig. 3:**
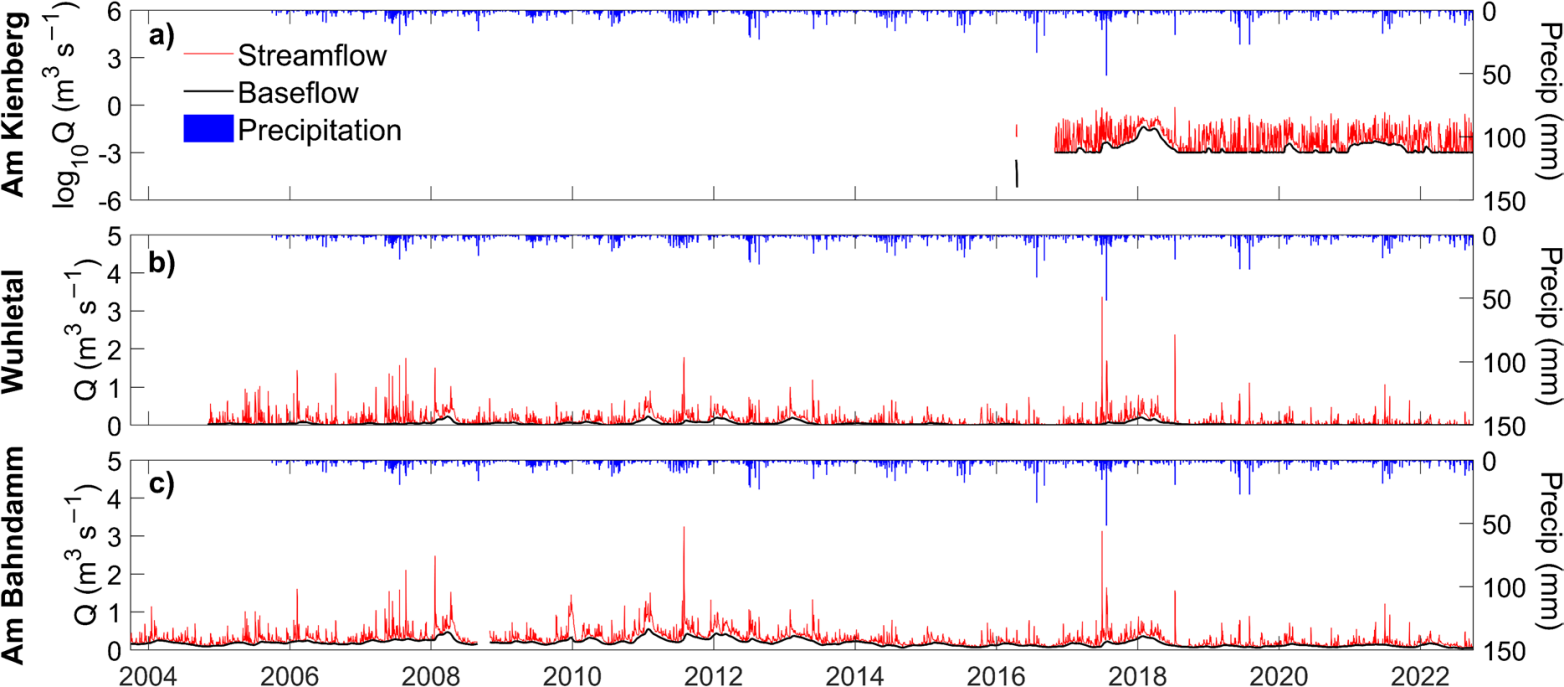
Daily time series of precipitation and runoff (showing hydrograph separation with HydRun) in the **a** upper catchment: *Am Kienberg* (base-10 log scale is used); **b** middle catchment: *Wuhletal*; and **c** lower catchment: *Am Bahndamm*

As reported by Smith et al. (2023) for the neighboring Panke catchment, Berlin experiences a warm, temperate maritime climate (*Cfb* in the Köppen classification). The average annual precipitation in the Wuhle catchment (2011–2020) was 590 mm, with frequent low-intensity winter precipitation and intense summer convective rainfall events yielding over 80 mm. Annual temperatures are moderate, averaging 10.3 °C, with seasonal monthly averages ranging from 1.2 °C in winter to 20 °C in summer. Recent protracted drought periods in Berlin and Brandenburg started in 2018 (Table 1, Fig. 3). These have resulted in low soil moisture levels in the summer, severely reduced groundwater recharge, and considerable vegetation stress across extensive areas (Haase & Hellwig, 2022; Kleine et al., 2020; Smith et al., 2020). As a result, stream flow in the area is becoming increasingly seasonal and many headwater streams have stopped flowing in summer (Kleine et al., 2021).

**Table 1:** Characterization of precipitation and stream flow components at the three gauging stations in the Wuhle catchment

Water year	Precip depth (mm)	Cumulative stream flow depth (mm)	Cumulative base flow depth (mm)	Cumulative storm flow depth (mm)	Base flow index (BFI)	Runoff coefficient	Storm runoff coefficient
Am Kienberg							
2017	739.6	18.8	1.2	17.6	0.06	0.03	0.02
2018	519.7	33.1	8.7	24.4	0.26	0.06	0.05
2019	546.4	11.1	0.8	10.3	0.07	0.02	0.02
2020	527.0	11.9	1.0	10.9	0.09	0.02	0.02
2021	600.5	14.7	2.4	12.3	0.16	0.02	0.02
2022	461.4	10.7	0.9	9.8	0.08	0.02	0.02
Wuhletal							
2008	588.3	71.0	28.3	42.7	0.40	0.12	0.07
2009	595.6	38.4	13.9	24.5	0.36	0.06	0.04
2010	705.8	47.3	16.0	31.4	0.34	0.07	0.04
2011	755.0	84.8	31.2	53.6	0.37	0.11	0.07
2012	636.3	69.9	31.5	38.3	0.45	0.11	0.06
2013	600.1	63.2	24.8	38.4	0.39	0.11	0.06
2014	539.1	34.7	12.4	22.4	0.36	0.06	0.04
2015	486.3	24.2	7.5	16.8	0.31	0.05	0.03
2016	626.2	28.2	5.2	23.0	0.18	0.05	0.04
2017	739.6	44.6	8.5	36.1	0.19	0.06	0.05
2018	519.7	80.0	36.2	43.9	0.45	0.15	0.08
2019	546.4	24.2	6.0	18.2	0.25	0.04	0.03
2020	527.0	19.5	3.0	16.5	0.15	0.04	0.03
2021	600.5	21.0	2.6	18.4	0.12	0.04	0.03
2022	461.4	12.5	1.8	10.7	0.15	0.03	0.02
Am Bahndamm							
2005	707.1	71.3	44.8	26.6	0.63	0.10	0.04
2006	482.7	85.0	56.3	28.7	0.66	0.18	0.06
2007	810.4	101.9	65.4	36.5	0.64	0.13	0.05
2008	588.3	112.0	72.2	39.8	0.65	0.19	0.07
2009	595.6	72.6	50.3	22.4	0.69	0.12	0.04
2010	705.8	115.8	67.5	48.4	0.58	0.16	0.07
2011	755.0	156.1	93.0	63.2	0.60	0.21	0.08
2012	636.3	136.6	87.8	48.9	0.64	0.21	0.08
2013	600.1	104.8	67.5	37.3	0.64	0.17	0.06
2014	539.1	71.0	45.0	26.0	0.63	0.13	0.05
2015	486.3	55.0	36.8	18.3	0.67	0.11	0.04
2016	626.2	56.0	34.7	21.3	0.62	0.09	0.03
2017	739.6	77.8	38.6	39.2	0.50	0.11	0.05
2018	519.7	93.5	59.7	33.8	0.64	0.18	0.07
2019	546.4	45.8	25.4	20.4	0.55	0.08	0.04
2020	527.0	51.5	32.1	19.4	0.62	0.10	0.04
2021	600.5	53.2	33.2	20.0	0.62	0.09	0.03
2022	461.4	46.7	26.7	20.0	0.57	0.10	0.04

Human occupation in the Wuhle valley goes back to the Paleolithic, with the earliest permanent settlements dating back to the thirteenth century when villages were founded and agriculture slowly intensified following extensive forest clearance (Geoportal Berlin / [Archeologic Sites], 2023, https://fbinter.stadt-berlin.de/fb/index.jsp). Wetland drainage became widespread in Brandenburg in the eighteenth and nineteenth centuries opening up new cultivation areas that had been intensively farmed in the twentieth century (Nützmann et al., 2000).

Berlin’s growth in the nineteenth century also brought major sewage management problems and associated risks to public health. In the 1860s, the *Hobrechtsplan* resulted in a canal system being developed to dispose of sewage and rainwater through infiltration and sewage field management (Hobrecht, 1884). Part of this involved disrupting the natural flow of the Wuhle and led to enhanced discharge and localized flooding. Subsequent interventions to mitigate these problems involved deepening, straightening, and regular maintenance of the river channel network (Fig. 1a and b).

Further changes occurred with the construction of the Falkenberg sewage treatment plant in 1984 and the creation of its artificial clean water drainage channel, the Neue Wuhle, which flows along the Alte Wuhle and converges with it south of the *Wuhleteich* in the middle catchment (Fig. 1b). After the closure of the treatment plant in 2003 (Möller & Burgschweiger, 2008), fears that the Wuhle would dry out sparked a significant catchment-wide restoration project, with the notable implementation of nature-based and technical solutions. Retention storage ponds were implemented as wetlands to capture high flows of the Wuhle. In 2014, 12 such ponds created an average potential storage capacity of 95,000 m^3^. More recently, four new ponds provided an additional storage volume of 27,700 m^3^ (ARGE Wasser, 2014). Additionally, groundwater abstractions from two water works may cause unintentional riverbank filtration; the maximum abstractions are currently of ~ 30.000 m^3^/d (equivalent ~ 0.7 m^3^/s) though the effects on the Wuhle are unknown.

Similar to other urban streams, wastewater management has evolved. In the north, formerly two sewage irrigation farms were operating – 1884/86 – 1969, treating in total ~ 70.000 m^3^/d (SenStadtUm, 1992), replaced by a smaller wastewater treatment plant (WWTP), operating until 2003 (~ 7.300 m^3^/d) (Möller & Burgschweiger, 2008). Some of the sewage irrigation farms were overbuilt with prefabricated buildings for housing. The discontinuation of the WWTP was expected to cause intermittent stream flow, decreasing groundwater levels in the Wuhle, and related endangerment of biotopes. Between 2006 and 2008, river restoration became more widespread (ARGE Wasser, 2014). This included removing weirs, morphologic improvements to the riverbed and banks, restoration of contaminated soils, and construction and restoration of retention basins leading to the re-wetting of formerly dry biotopes, achieved by bypassing the Wuhle with a side channel and using both river and rainwater as water sources (ARGE Wasser, 2014). Most of the sewer system is separated (Fig. S1), with wastewater being transported outside of the catchment, while rainwater either infiltrates locally as a result of low impact development (LID), notably including urban green spaces, or is routed to the streams via storm drains, attenuated in some areas by storage and/or retention structures (Geoportal Berlin / [Sewer System], 2017, https://fbinter.stadt-berlin.de/fb/index.jsp).

Today, the upper catchment in the State of Brandenburg remains mostly rural, the land being used for agriculture, with some patches of forest and wetland areas (Fig. 1c, Table S1). The middle and lower catchments lay within Berlin’s city limits (ca. 57 km^2^) and are highly urbanized (Fig. 1c), accounting for most of the impervious areas (Fig. 1d). Approximately 14% of the catchment is sealed and connected to stormwater drains.

### Datasets

#### Maps

Catchment maps were produced in QGIS (version 3.28.7) based on publicly available official datasets (FIS-Broker / Geoportal, fbinter.stadt-berlin.de). The delineation of sub-catchments for the rainfall-runoff modeling (Fig. 1b) was based on the micro-catchments defined by the *Landesamt für Umwelt Brandenburg* (LfU) and provided by the *Landesvermessung und Geobasisinformation Brandenburg* (LGB) (2012, revised in 2021). Topography (200-m resolution DTM), watercourses, and other major bodies of water were mapped based on the lakes and waterways network datasets provided by the same offices. Land use and vegetation characteristics, as well as the percentage of impervious areas, were mapped based on the Urban Atlas 2018 (European Environment Agency, Copernicus Land Monitoring Service).

#### Climate and stream flow data

Climatic datasets provided by the German Weather Service (DWD, 2023) for the stations in and around the catchment were used to characterize weather conditions. For stream flow, the publicly available discharge datasets provided by the Berlin Senate Department of Environment, Transport and Climate Protection (SenUMVK, 2022)—Am *Kienberg (Hellersdorfer Graben)*, *Wuhletal* and *Am Bahndamm* gauging stations (Fig. 1b)—were used.

#### Water chemistry, stable isotopes, and physical parameters

The stream water chemistry and stable water isotope datasets were analyzed for rain and stream waters at the Leibniz Institute of Freshwater Ecology and Inland Fisheries (IGB) in SE Berlin. Rainfall was sampled daily at IGB and stream water was sampled at weekly intervals at the *Alte Försterei* sampling station since 2019 for stable water isotopes and 2021 also for water quality parameters (Fig. 1b). Groundwater samples were collected from several of the wells shown in Fig. 2 as part of a city-wide synoptic sampling campaign conducted in 2018 (Kuhlemann et al., 2020, 2021). Synoptic surveys in 2022–2023 sampled the stream and 7 storm retention ponds.

Physicochemical parameters were measured during field sampling activities using a WTW MULTI 3630 IDS Set for pH (SenTix940, precision ± 0.0004), dissolved oxygen (DO; FDO925, precision ± 0.5% DO), temperature (precision ± 0.2 °C), and electrical conductivity (EC; TetraCon925, precision ± 0.5%). For the analysis of dissolved chloride (Cl^−^), nitrogen-nitrate (NO_3_^−^-N), and sulfate (SO_4_^2−^) water samples were collected in 2-mL Eppendorf tubes and analyzed by ion chromatography (Metrohm CompactIC, conductivity detection after chemical suppression). For dissolved inorganic carbon (DIC), water was collected in 20-mL glass vials and analyzed with a Shimadzu TOC-L Total Organic Carbon Analyzer with an analytical precision of < 3% and for dissolved metals (Al, B, Ca, Fe, K, Mg, Mn, Na, P, S, Si) water was collected in 15-mL tubes, acidified with 150 µL of 2 M HCl and analyzed by inductively coupled-optical emission spectroscopy (ICP-OES, Thermo Scientific iCAP 6300).

For stable water isotope sampling, stream water was filtered through an acetate filter (0.22 µm cellulose acetate) into 1.5 mL vials (LLG Labware) on site. Daily precipitation isotope samples were collected in the east of Berlin at the IGB using an ISCO 3700 autosampler (Teledyne Isco, Lincoln, USA). All water samples (stream and precipitation) were analyzed using Cavity Ring-Down Spectroscopy using a Picarro L2130i Isotopic Water Analyzer (Picarro Inc., Santa Clara, CA). The standards of the Vienna Standard Mean Ocean Water (VSMOW) of the International Atomic Energy Agency (IAEA) were used for calibration. The analytical precision of liquid analyses had a standard deviation of 0.03‰ for δ^18^O and 0.13‰ for δ^2^H.

### Statistical analysis

A composite dataset including all basic water quality and chemistry variables, as well as stable isotope data from all sampling campaigns, and available discharge data, were normalized in the form of standard scores before being subjected to a principal component analysis (PCA). A pairwise correlation analysis at a 99% confidence level was carried out for all water quality, isotope, and discharge variable pairs.

### Hydrograph separation (HydRun)

Time series of base flow for the three gauging stations in the catchment (Fig. 1b) were produced using HydRun, a MATLAB-based package for event-based rainfall-runoff hydrograph analysis (Tang & Carey, 2017). HydRun’s base flow separation method implements a recursive digital filter technique (Nathan & McMahon, 1990). This technique decomposes high-frequency and low-frequency signals, commonly associated with storm flow and base flow, respectively (Arnold et al., 1995; Chapman, 1999; Eckhardt & Arnold, 2001; Nathan & McMahon, 1990). The input includes stream flow and rainfall data, ideally of hourly or finer resolution, to match a precipitation event with the corresponding discharge response. HydRun produces base flow time series that are more realistic in comparison with conventional manual hydrograph separation methods (no zigzag behavior, as in works by Sloto & Crouse, 1996; and Davie, 2008).

In this study, we analyzed publicly available hourly discharge and precipitation data. We chose a filter coefficient of 0.995, as we found lower values to be ineffective in filtering out high-frequency runoff peaks. The filter was set to pass through the hydrograph 4 times. The resulting base flow and storm flow time series were used to estimate the Base Flow Index and the Storm Runoff Coefficient, respectively.

### Estimation of the Mean Transit Time (MTT) and the Young Water Fraction (YWF)

To characterize flow paths and associated transit times in the catchment, we estimated the Mean Transit Time (MTT) and the Young Water Fraction (YWF). The methodology employed in this study is an extension of preliminary research conducted in the same area but with a more extensive time series (Kuhlemann et al., 2022). The MTT represents the average time elapsed between the entry of a water molecule into a catchment (e.g., rainfall) and its exit from the catchment (Benettin et al., 2022). The calculation of the MTT involved the use of a lumped convolutional model based on the gamma distribution (McGuire & McDonnell, 2006), a family of probability distributions that is versatile and requires the estimation of only two parameters: *α* and *β*. The *α* parameter determines the shape of the distribution, capturing the effect of catchment characteristics such as soil cover and drainage density, which are associated with the fast runoff component in an urban catchment. The *β* parameter scales the distribution, capturing long-term memory effects (Hrachowitz et al., 2010). This makes the gamma modeling approach a more parsimonious choice relative to dual reservoir models that involve an additional and often unidentifiable partitioning parameter, both of which have been successfully used to approximate Transit Time Distributions (TTDs) in urban catchments (Soulsby et al., 2014). In this study, optimal *α* and *β* values were determined based on the maximization of the Kling-Gupta Efficiency (KGE) between measured and estimated stream oxygen-18 signals using a Monte Carlo Markov Chain (MCMC) method.

Additionally, the YWF was estimated by first fitting a sinusoidal wave to both the stream water and the precipitation oxygen-18 data derived from collected water samples, and then calculating the ratio between the two (as in Kirchner, 2016a, 2016b). The Iteratively Re-weighted Least Squares (IRLS) R script by Kirchner and Knapp (2020, version 1.4) was applied to enhance the robustness of fitting and mitigate the impact of outliers. In general, a relationship between the optimized MTT parameterization and the water age of the YWF can be established based on the shape parameter of the fitted gamma distribution, i.e., the *α* parameter. For instance, an optimized value of *α* = 0.5 signifies that “young water” is younger than 1.7 months; *α* = 1.0, that it is younger than 2.3 months; and *α* = 1.5, that it is younger than 2.7 months.

### Rainfall-runoff modeling

A semi-distributed rainfall-runoff model of the Wuhle catchment was implemented using the Hydrologic Engineering Center—Hydrological Modeling System (HEC-HMS, Fig. S2), developed by the US Army Corps of Engineering for dendritic catchments (Feldman, 2000). A detailed model description is included in the Supplementary Information (SI) section. Sub-catchments were delineated (Fig. 1b) to make the best possible use of available discharge data from existing gauging stations (upper, *Am Kienberg*; middle, *Wuhletal*; lower, *Am Bahndamm*) and stream isotope datasets (water sampled at sampling site *Alte Försterei*). The model was parameterized based on differences in regional land cover characteristics (Fig. 1c–d). In such a relatively flat area overlying a regional aquifer, there is considerable uncertainty over the catchment area defined by a DTM and the contributions to stream flow from the underlying aquifer systems (Moore et al., 2020). Modeled sub-catchments were also delineated based on urban features such as highways and drainage ditches that modify natural catchment boundaries.

Model calibration used a Latin Hypercube Sampling (LHS) design to generate a near-random sample of 23 parameter values for each of the 11 sub-catchments from within plausible ranges based on prior work in the neighboring Panke catchment by Smith et al. (2023) (Table S2). A total of 150.000 simulations were carried out on Cirrus, a Linux-based High-Performance Computing Cluster at the Humboldt University in Berlin. The model was calibrated on the measured stream flow at gauging station *Am Bahndamm*, and optimized on NSE using 12 hourly time steps. The latter was a compromise based on the need to capture both short (sub-daily) and long (~ decadal) response timescales with computational efficiency.

Isotope datasets were used as an independent auxiliary measure to test the realism of the models in terms of flow path partitioning via an additional isotope mixing model (Figure S1b). Mixing of stable isotopes (δ^2^H and δ^18^O) and water ages were computed for the fluxes and storages from calibrated HEC-HMS results. Additional effects of passive storage on damping the isotopic dynamics were incorporated using an amount-weighted approach (as in Smith et al., 2023), assuming a complete and uniform mixing for each time step as with other isotopic modeling approaches (Ala-Aho et al., 2017; Kuppel et al., 2018). At the end of each time step, one time step was added to each average water age in storage to account for the aging of water. Because in-stream evaporative fractionation is negligible in Berlin (see results for the Panke stream by Smith et al., 2023), these effects were not considered within the isotope mixing module.

## Results

### Hydrological dynamics

Figure 3 shows the time series of precipitation and stream flow for the three gauging sites on the Wuhle between 2004 and 2022, and the decomposition of stream flow into its base flow and storm flow components. This period saw marked variability in annual precipitation ranging from 461 mm in 2022 to 810 mm in 2007 (Table 1). In common with the rest of Berlin and Brandenburg, most years since 2017 have seen rainfall deficits compared to the long-term average. Most rainfall was low in intensity (typically < 10 mm/d) with the heaviest rain (> 20 mm/d) mostly restricted to convectional storms in the summer.

The Wuhle is a “flashy” stream and was highly responsive to significant rainfall events during the study period, consistent with the effects of urban storm drainage from impervious surfaces. This flashiness increased downstream as the urban area became more extensive (Table 1, Fig. 3). However, the multiple flood storage ponds in the catchment mitigated runoff peaks when antecedent storage was available, so runoff peaks did not necessarily increase proportionately to rainfall inputs. Moreover, most of the highest flows tended to be in response to summer convectional storms.

Despite the flashy runoff regime, evapotranspiration accounted for 80–90% of rainfall and base flows were the dominant component of stream flow in terms of runoff volumes in the lower catchment (Table 1). Annual runoff coefficients were low; ranging from < 5% in the upper catchment to ~ 10–20% in the lower catchment (Table 1). This likely reflects greater evapotranspiration and groundwater recharge, and less direct storm runoff in the upper non-urban catchment. Annual runoff has been exceptionally low at each gauging point since the drought of 2018 (~ 50% of the annual mean of previous years on record).

The base flow components of annual runoff also increased downstream with the BFI typically increasing from <  ~ 0.2 in the upper catchment, to ~ 0.2–0.4 in the mid-catchment, to 0.4–0.6 in the lower catchment (Table 1). Seasonally varying groundwater inputs in the lower catchment were apparent and the groundwater component of base flow has likely been decreasing since 2017 in response to the drought years.

**Fig. 4:**
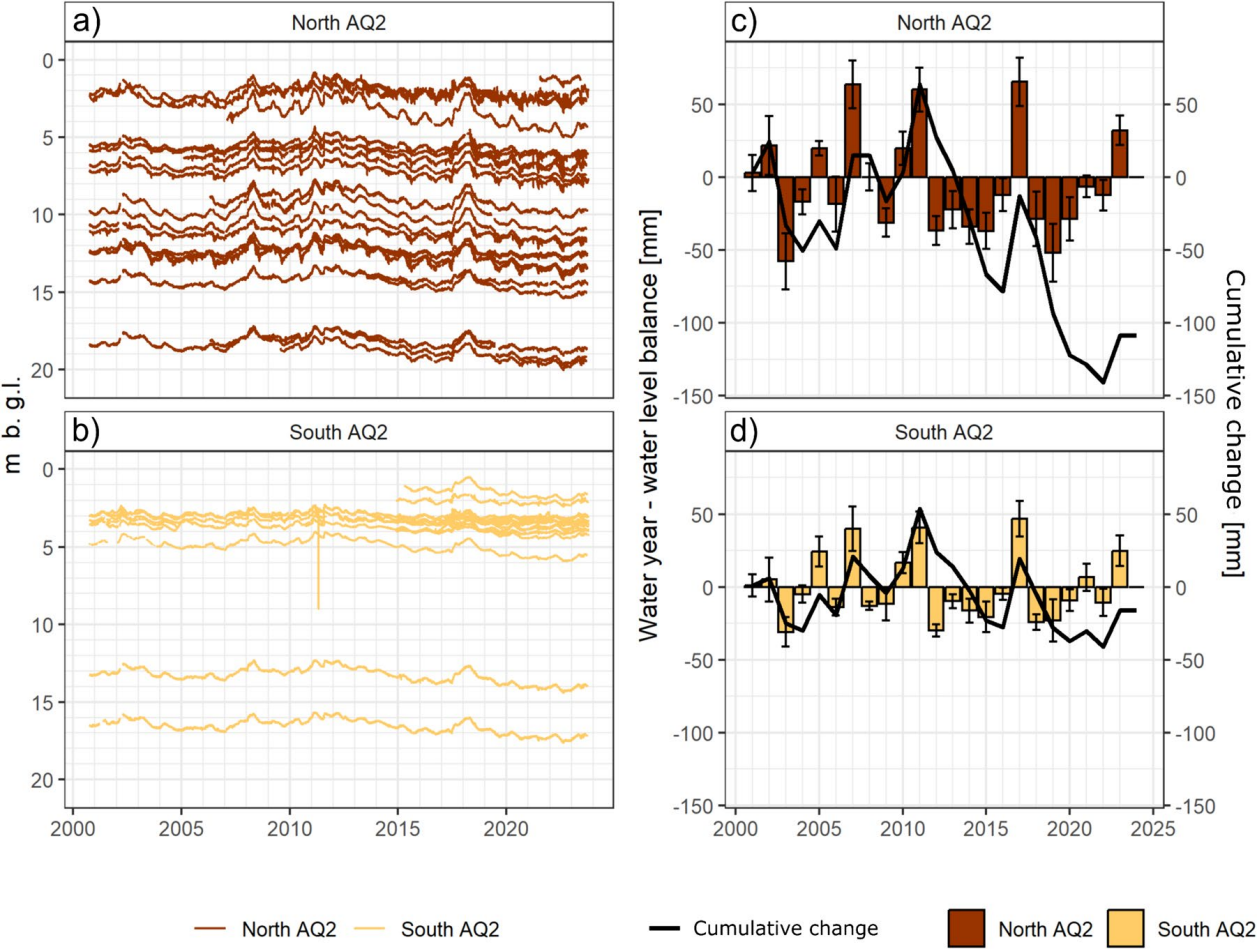
Groundwater levels in the wells located in **a** the confined (North AQ2) and **b** unconfined (South AQ2) aquifers of the Wuhle Valley; and the average and cumulative annual changes in groundwater levels in **c** the North AQ2 and **d** the South AQ2

These temporal dynamics and recent changes in the flow regime were consistent with a general response to regional groundwater levels. The time series of individual wells in the confined AQ2 aquifer in and around the northern part of the Wuhle catchment and the unconfined AQ2 aquifer in the south are presented in Fig. 4a and b. While the water table is closer to the surface in most wells in the unconfined aquifer in the south, there is variation in depth to the water table depending on locations. However, most wells displayed clear seasonality of late winter peaks and late summer minima. The effects of the wetter years of 2007, 2010–11, and 2017 on groundwater levels were also apparent. Most wells exhibited a gradual decrease since the wet years of 2010 and 2011, with a marked decline since 2017/18. These patterns are clearer when the annual average change in water table level is estimated for each year consistent with a direct link between climatically driven reduced recharge, lowering water tables, and a decrease in stream base flows (Fig. 4).

### Dynamics of stable water isotopes

**Fig. 5:**
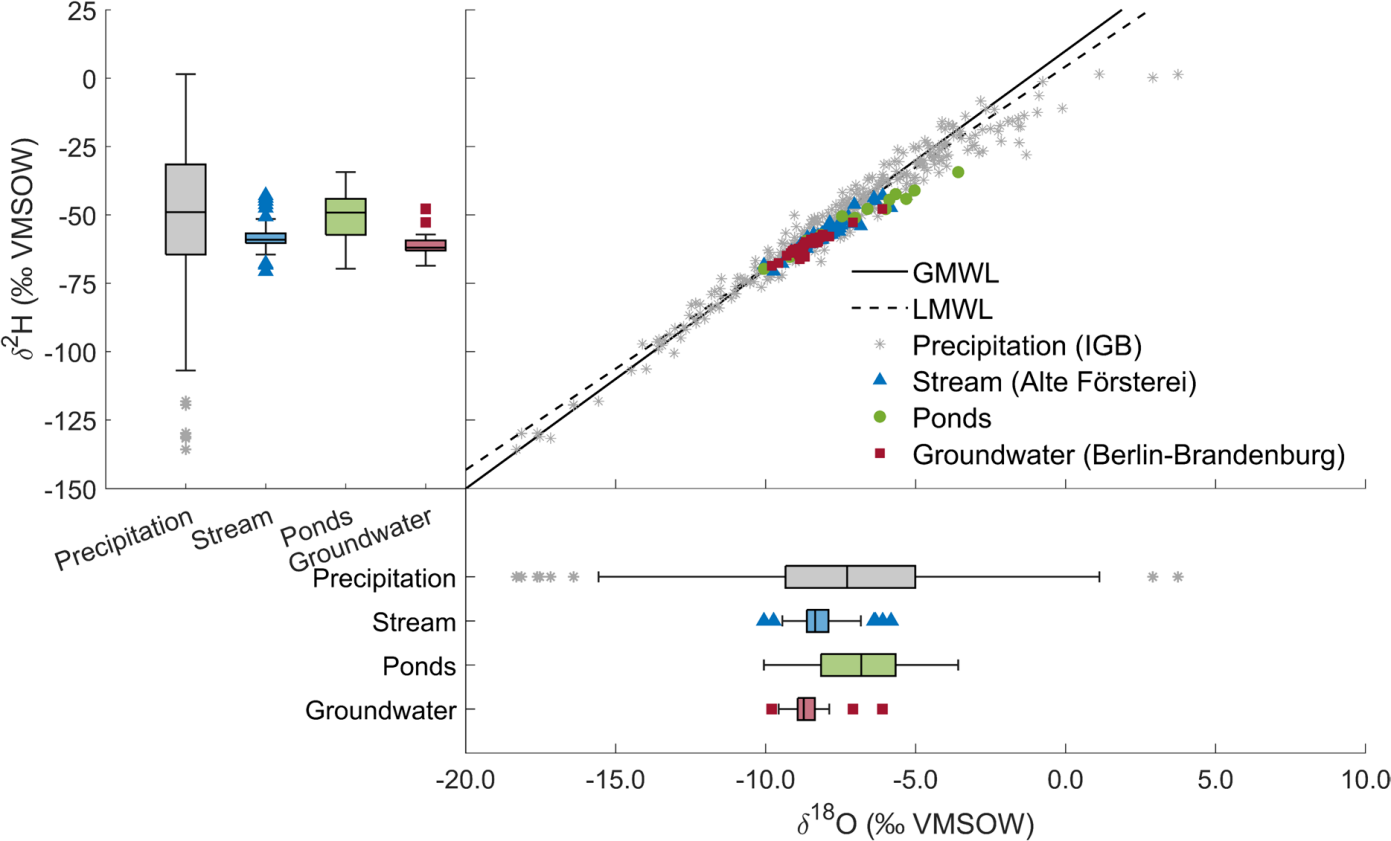
Dual isotope plot showing deuterium (δ^2^H) and oxygen-18 (δ^18^O) ratios in precipitation, stream, pond, and groundwater samples collected in the Wuhle catchment

Stable isotopes of water in precipitation close to the Wuhle catchment (~ 5 km) showed marked variability (Fig. 5). Much of this is related to seasonality, with rainfall that is more enriched in heavy isotopes in summer and more depleted in winter. Nevertheless, within seasons, day-to-day variability in the isotopic composition of rainfall was also marked and, in summer convective events, samples were enriched in heavy isotopes that plotted below the Local Meteoric Water Line (LMWL) indicating atmospheric recycling of moisture (Fig. 5).

**Fig. 6:**
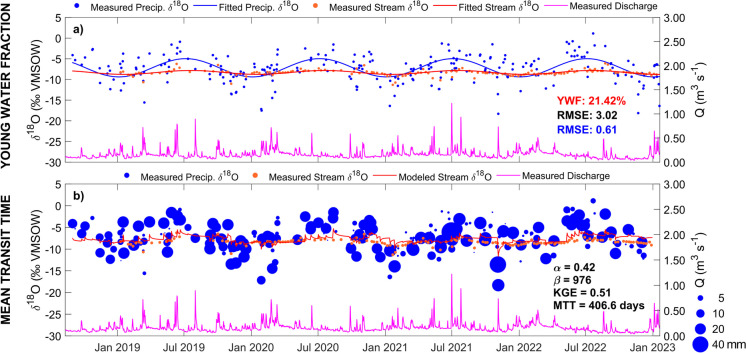
Time series of δ^18^O in rainfall (*IGB*) and runoff (*Alte Försterei*), measured precipitation (*Buch*), and gauged discharge Q (*Am Bahndamm*) showing fitted estimates for the Wuhle catchment of **a** the Young Water Fraction (YWF), and **b** the Mean Transit Time (MTT)

Isotopic variability in stream water was highly damped compared to rainfall (Fig. 5). The average composition was close to that of groundwater, though groundwater was more stable and isotope ratios more negative, consistent with the predominance of winter recharge (Fig. 6). During high flow events, the stream water isotopic signal tended to move in the direction of recent rainfall, though the effect was most pronounced in larger, more intense events (Fig. 6). This would be consistent with the effects of urban storm drains routing rainfall rapidly into streams. However, the effects were non-linear depending on the role of urban storm retention ponds, some of which are operated via weirs and return water into the stream when certain storage thresholds are surpassed.

Despite the relatively coarse weekly sampling, overall estimates of the YWF produced by the isotope-based regression model (Fig. 6a) were around 20% and consistent between years. From the limited direct influence of storm runoff, we infer that isotopically well-mixed groundwater provided the dominant source of stream flow over the study period. This isotope-based analysis also supported the hydrometric analysis (Fig. 3), which indicated that the high base flow component was sustained by groundwater explaining the relatively low runoff coefficients at both the annual and storm event scale.

The MTT of the stream derived from fitting a gamma function to the rainfall-runoff isotope time series was ~ 1.1 years, with an optimized alpha value of 0.41. This, in the context of the YWF, again implies contributions of older groundwater water in the long tail of the travel time distribution. Sampling of some of the storage ponds indicated evaporative effects in water retained in off-line storage though effects on stream isotopes were not detected.

### Water quality dynamics

**Fig. 7:**
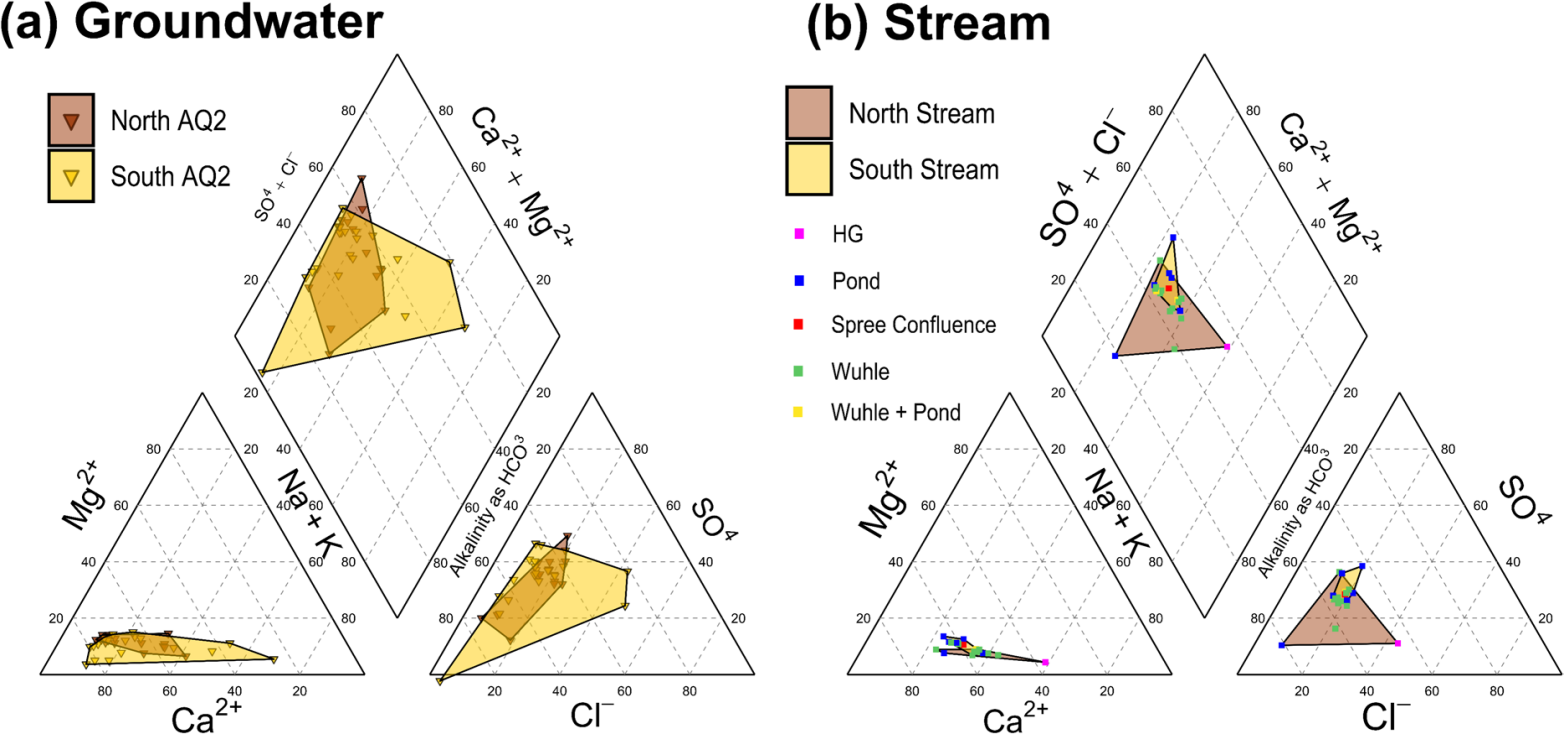
Major ion chemistry of **a** groundwater; and **b** stream flow in the Wuhle catchment plotted as Piper diagrams

Table 2 summarizes the hydro-chemical characteristics of the Wuhle stream at the catchment outlet. The stream was mildly alkaline with a mean pH of 7.7 and a high solute load indexed by its high electrical conductivity (~ 823 ± 108 µS cm^−1^). The stream had, at times, elevated levels of typical urban pollutants such as P, B, and Cl (max values were 0.22, 0.02, and 152.8 mg L^−1^, respectively), though overall levels of many pollutants such as NO_3_-N were low (0.33 ± 0.20 mg L^−1^). This is consistent with the dominance of groundwater with a long residence time, as demonstrated by the major ion chemistry, which is summarized in a Piper plot in Fig. 7. Like groundwater (Fig. 7a), the stream (Fig. 7b) was predominantly dominated by HCO_3_^–^ and Ca^2+^ ions. The stream network samples had less heterogeneity (higher spread of points in Fig. 7a than in Fig. 7b) and only limited spatial variability in chemical composition compared to that of groundwater samples (less overlap between North and South polygons in Fig. 7b relative to Fig. 7a). The variability in groundwater composition is particularly high in the unconfined aquifer south of the catchment (higher spread of points and larger polygons in Fig. 7a than in Fig. 7b), where shallow groundwater is vulnerable to surface sources of pollution (e.g., from road salts). Consequently, SO_4_^2–^ and Cl^–^ became more important anions downstream.

**Table 2:** Summary of WQ parameters. Mean, standard deviation, maximum and minimum values (2021–2023)

Variable (units)	Mean (2021–2023)	Standard deviation (2021–2023)	Maximum (2021–2023)	Minimum (2021–2023)
pH	7.70	0.22	8.82	7.32
EC (µS cm^−1^)	822.88	108.02	1133.00	463.00
Temperature (°C)	10.34	6.14	23.10	− 0.40
O_2_% (saturation)	58.77	26.45	138.10	11.70
O_2_ (concentration)	6.61	2.95	15.55	1.45
δ^2^H (‰ VMSOW)	− 58.86	3.87	− 42.91	− 68.31
δ^18^O (‰ VMSOW)	− 8.36	0.59	− 6.10	− 10.06
Ca (mg L^−1^)	116.54	17.45	146.50	61.90
Mg (mg L^−1^)	12.91	2.31	17.54	5.70
Na (mg L^−1^)	32.12	7.85	84.02	18.30
K (mg L^−1^)	6.85	0.76	9.10	4.48
DIC (mg L^−1^)	58.39	9.79	74.00	28.50
SO_4_^2−^ (mg L^−1^)	109.26	22.96	205.30	11.80
Cl^−^ (mg L^−1^)	54.61	14.12	152.80	33.10
NO_3_^−^-N (mg L^−1^)	0.33	0.20	0.93	0.05
Si (mg L^−1^)	6.69	1.48	9.70	3.98
P (mg L^−1^)	0.05	0.04	0.22	0.01
Fe (mg L^−1^)	0.05	0.04	0.22	0.01
Mn (mg L^−1^)	0.09	0.06	0.28	0.01
Zn (mg L^−1^)	0.01	0.02	0.17	0.00
B (mg L^−1^)	0.06	0.01	0.08	0.02
Al (mg L^−1^)	0.01	0.00	0.02	0.00

**Fig. 8:**
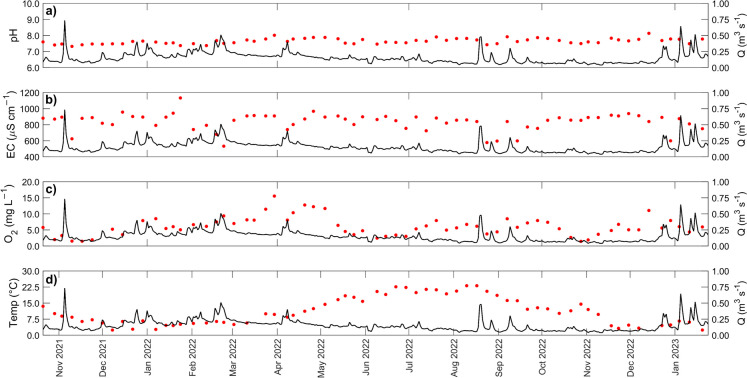
Basic water quality parameters in weekly samples of the Wuhle stream (*Alte Försterei*): **a** pH; **b** electrical conductivity (EC); **c** dissolved oxygen; and **d** temperature

In addition to the stream and groundwater, the Piper diagram incorporates data from other surface waters such as storm detention ponds and ditches, e.g., the Hellersdorfer Graben (HG), which drains the sub-catchment SC7 (Fig. 1b). In some cases, these differed substantially from the northern-confined aquifer, as detention ponds most likely reflect the influence of rainfall rather than groundwater. In terms of flow-related variability, pH tends to be higher in summer, with higher flow events associated with slight depressions in pH (Fig. 8). This dilution in events is more pronounced for electrical conductivity (EC), consistent with the isotopic data that indicates a shift between stream flow being dominated by more concentrated groundwater and more diluted rainfall. Stream water temperature and O_2_ showed similar seasonal patterns, as expected, given the effect of temperature on the solubility of O_2_. However, it is notable that O_2_ levels are low for prolonged periods during the summer.

**Fig. 9:**
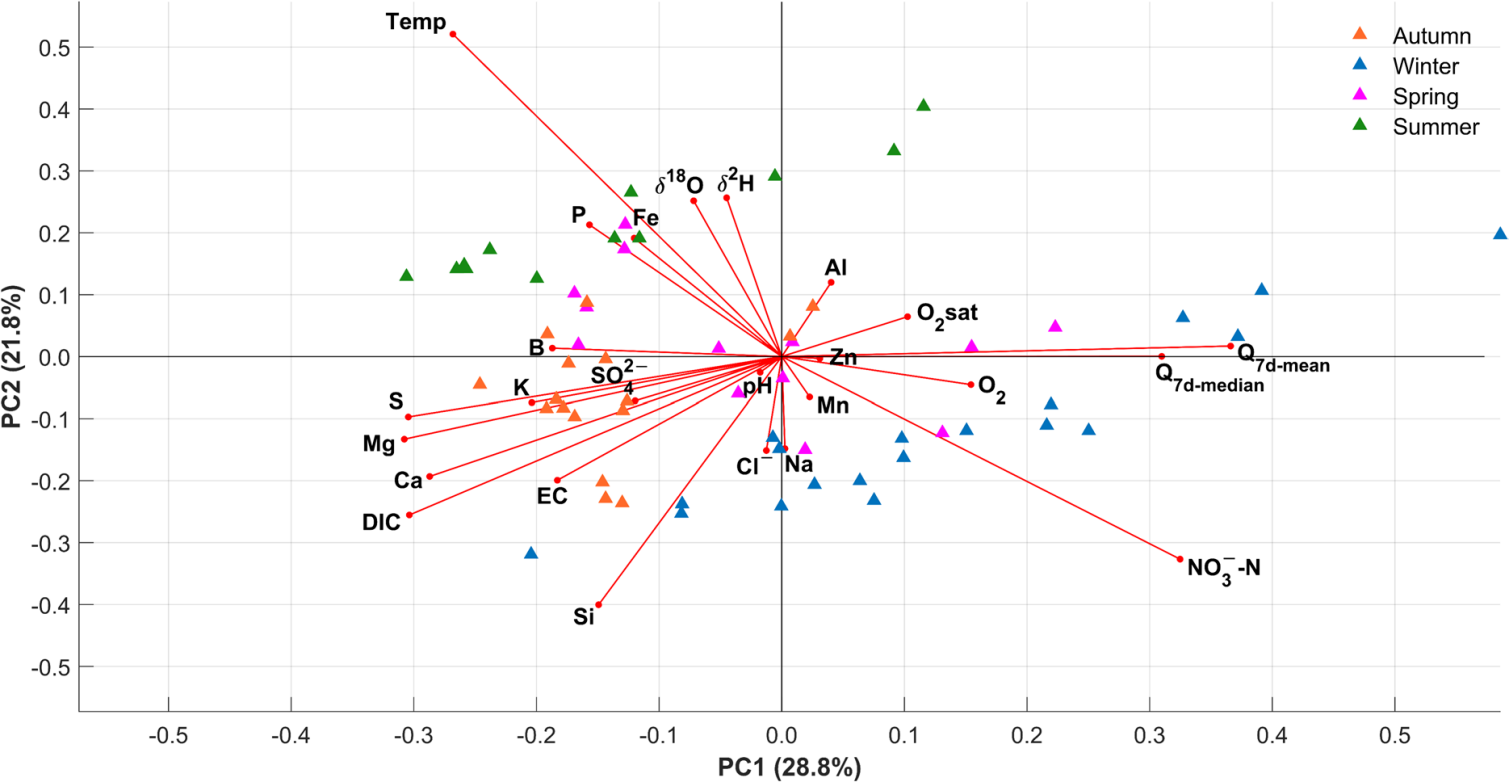
Principal components analysis of Wuhle stream water chemistry with individual measurements and samples collected at sampling site *Alte Försterei* (2021–2023) and classed according to season

The water quality data are plotted as a PCA in Fig. 9. As reflected in Fig. 8, flow had a strong influence on many water quality parameters as a direct consequence of dilution. Accordingly, metrics of stream flow showed strong positive loadings on axis 1 of the PCA. Dissolved O_2_, NO_3_^–^-N, Zn, and Mn also showed similar loadings, consistent with increased concentrations as flows increase. In contrast, strong negative loadings on EC, base cations, DIC, and SO_4_^2–^ were consistent with decreased concentrations at higher flows.

The influence of seasonality was evident in the positive loadings on temperature, which aligned with increased isotope ratios, P, and Fe. In contrast, Na^+^ and Cl^–^ showed negative loadings on axis 2, indicating different controls likely reflecting winter contamination from road salts.

### Rainfall-runoff modeling

**Fig. 10:**
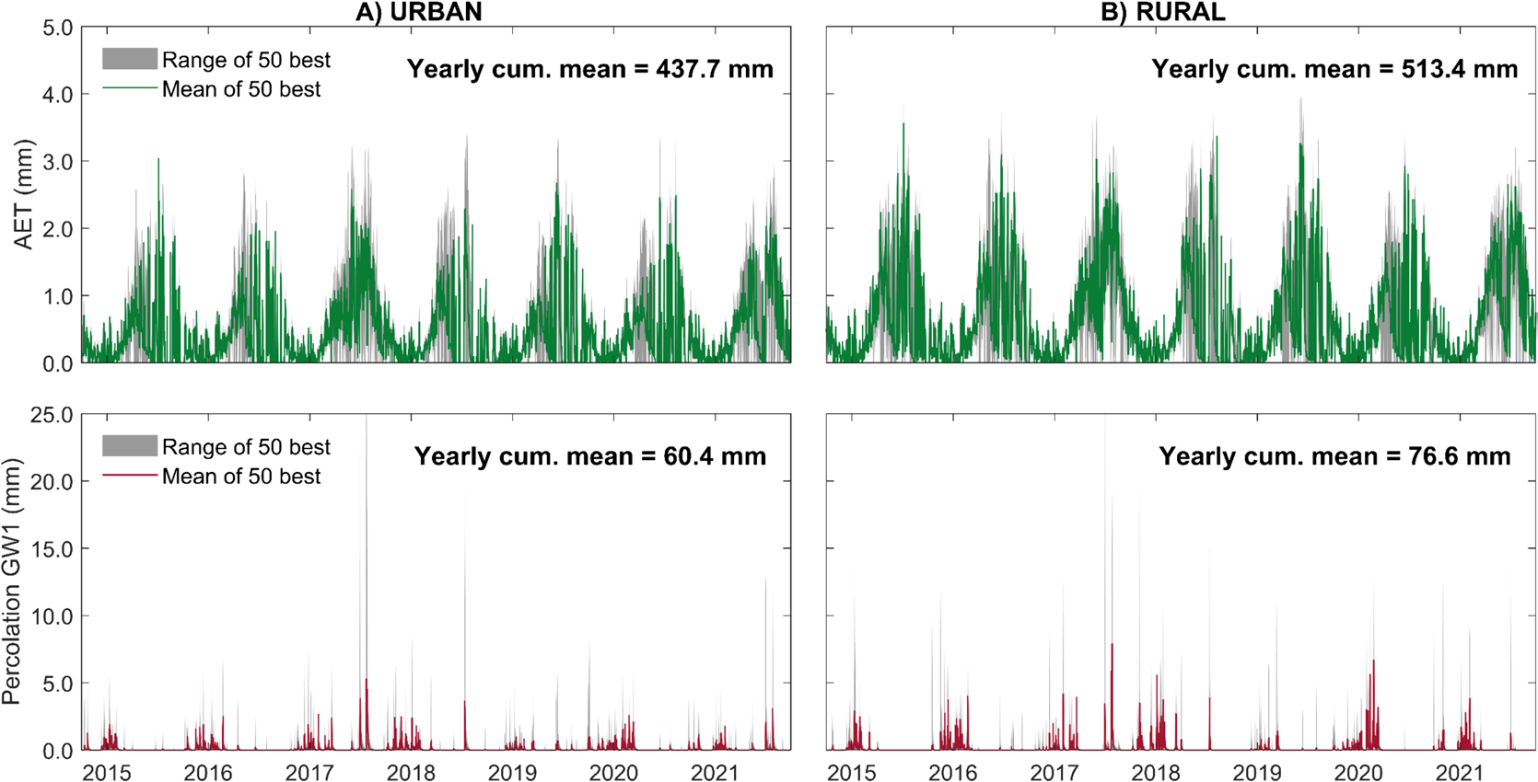
Modeled daily evapotranspiration and groundwater recharge for the **A** urban (SC2); and **B** rural (SC7) areas within the model domain

**Fig. 11:**
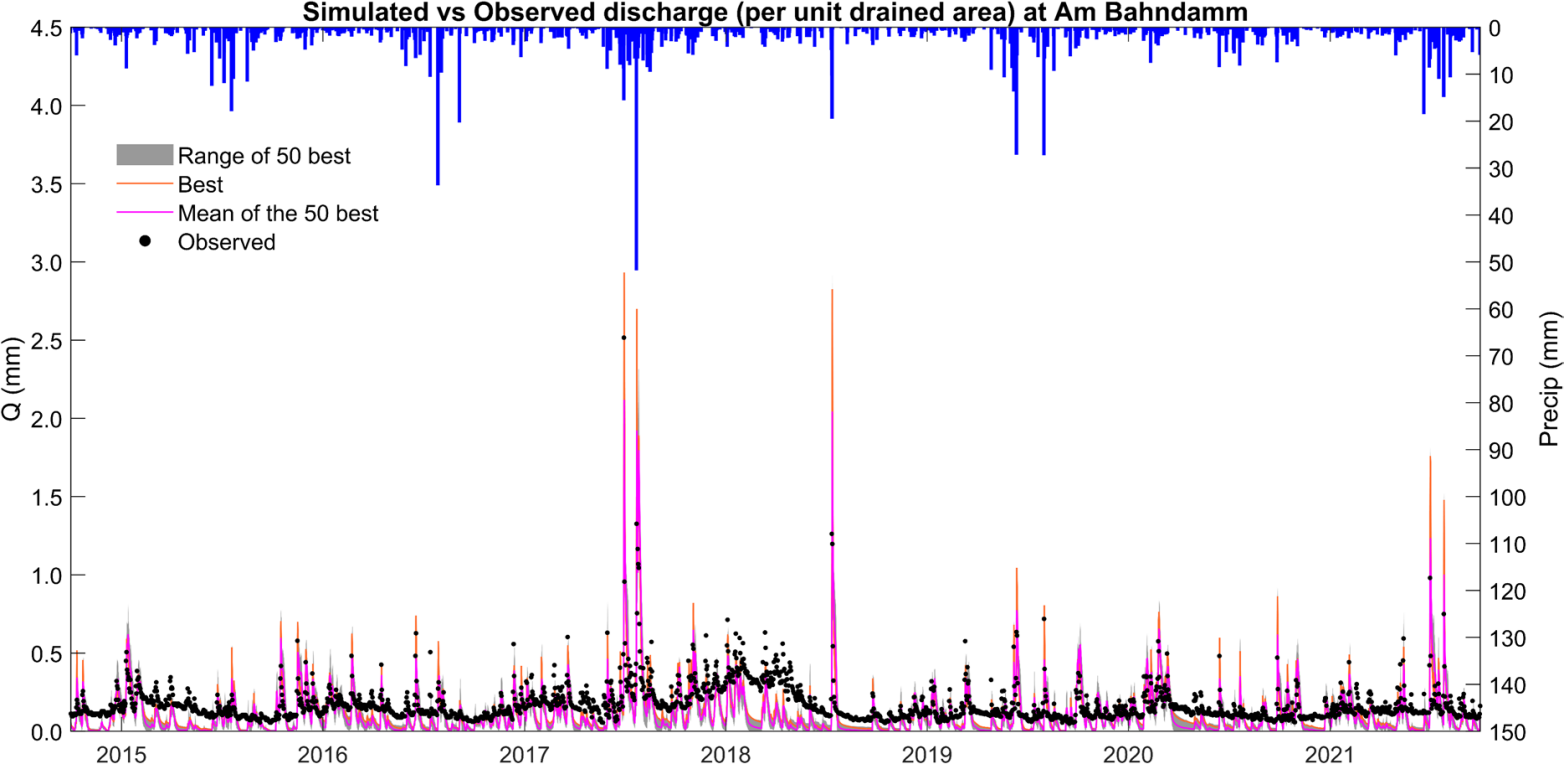
Simulated and observed discharge at *Am Bahndamm* (lower catchment) with uncertainty bands. Precipitation data is from the *Ahrensfelde* weather station in the upper catchment

**Fig. 12:**
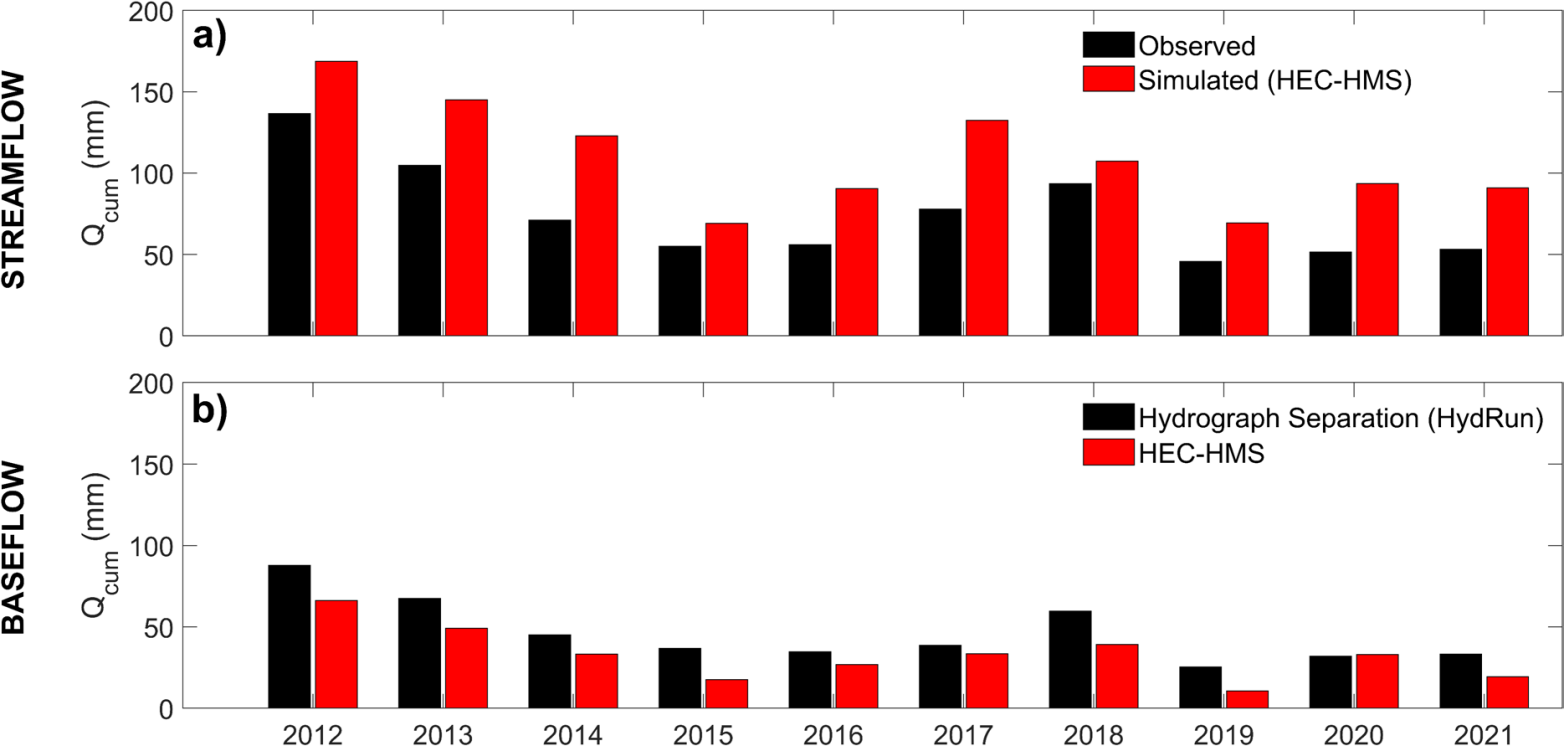
Measured (gauging station *Am Bahndamm*) and simulated annual cumulative stream flows and base flows for the lower catchment, derived from **a** the HydRun digital filter technique for hydrograph separation, and **b** rainfall-runoff modeling with HEC-HMS

HEC-HMS was calibrated to the catchment outflow to simulate runoff. The overall water balance of the catchment was captured, with higher annual ET and groundwater recharge from non-urban areas (Fig. 10a–b) which aligned with the increasing runoff coefficients downstream (Table 1). Interannual differences in ET and recharge also broadly captured the effects of the recent drought years. However, while the calibration of the model according to different efficiency statistics could produce a reasonable simulation of either flood peaks or base flows; balanced calibration of the model to adequately simulate both was only partly successful (Fig. 11). Such a “balanced” model tended to overpredict the largest peak flows (typically by 20–30%) and underpredict the lowest base flows, and it only yielded modest performance statistics from conventional objective functions, as well as overarching uncertainty over the actual catchment area. This likely reflects the combined effects of underpredicted infiltration due to the underestimation of surface storage in natural terrain depressions and water retention ponds, as well as their role in truncating peak runoff. Moreover, although the non-stationary influence of hydroclimate on annual base flows was captured similarly to the hydrograph separation method, flows were generally overestimated (Fig. 12) and the partitioning of runoff pathways was unable to reproduce the stream isotope signal (shown in Fig. 6), indicating insufficient incorporation of storage and mixing (Fig. S3).

## Discussion

### Enhancing understanding of complex urban hydrology

Urban streams drain complex catchments which integrate the effects of landscape heterogeneity from impermeable and permeable surfaces, with the respective influences of engineered drainage and more natural hydrological pathways through urban green spaces. While groundwater recharge can increase in an urban environment where leaking pipes and sewer systems can replenish groundwater (Lerner, 1990, 2002; Minnig et al., 2018), sealed surfaces and urban drainage more generally decrease recharge. In addition, urban green spaces ecohydrologically partition water between evapotranspiration losses (that can contribute to off-setting the urban heat island effect from latent heat transfers) and groundwater recharge (that retains water in the urban environment and can enhance stream flows) (Gillefalk et al., 2021). These complex interactions, including storm drainage from sealed surfaces, commonly cause urban streams to more frequently exceed extreme low or high flow thresholds (Debbage & Shepherd, 2018), though, in some cases, enhanced low flow conditions can occur (Chu et al., 2013). These sometimes-contradictory impacts resulting from interactions between the two “natural” and “artificial” flow systems in cities are poorly understood and are yet increasingly important for the management of urban water resources (Oswald et al., 2023). Such contradictions likely reflect the wide range of potential impacts that result from geographical differences in environmental conditions (climate, geology, topography, etc.) and the precise characteristics of the built environment (history, density, engineered infrastructure, proportion of green spaces, etc.) in contrasting urban settings.

While traditional urban hydrology focused on providing an evidence base for planning and managing urban drainage and flood alleviation, understanding recharge and generation of base flows are increasingly important in the face of challenges posed by climate change such as drier, warmer catchments. These challenges are becoming a reality and forcing a major rethinking of water management strategies in Berlin and other cities (Creutzfeld et al., 2021; SenUMVK, 2022). The hydrometric, isotopic, and hydro-chemical data from the Wuhle show the complementary effects of these interactions between more “natural” and engineered hydrological systems. Concerning our first specific research aim of characterizing the hydrology of the Wuhle, we have identified base flows in the catchment to be groundwater-dominated; the seasonal base flow component of the stream is synchronous with the annual cycle of groundwater recharge in winter and summer drawdown. Moreover, the long-term groundwater responses correlate well with non-stationarity in the stream base flow component. This is evident in the groundwater/stream flow highs in the wet 2009–2011 period, as well as in 2017, and the drier 2012–2016 and 2018–2022 periods. Regarding our second specific research aim of using isotopic, water quality, and hydrometric data, we have seen that its integrated interpretation supports these findings, mirroring the strong groundwater dominance in non-urban streams draining similar but rural catchments elsewhere in the German State of Brandenburg, which surrounds Berlin (Smith et al., 2021). Such groundwater is predominantly derived from unconfined aquifers and is relatively young. Recent tritium-based dating yields average base flow age estimates of around 5 years (Ying et al., 2024); this is of a similar order of magnitude to the MTT-derived ages of > 1 year, particularly when considering that this includes the influence of a 20% new water fraction, mainly derived from urban storm drains. These shallow groundwater influences contrast with those of the much older water in the deeper aquifer systems that are prevalent in Berlin-Brandenburg (Bednorz & Brose, 2017; Massmann et al., 2009). As in other parts of the world, it seems that these older waters have only limited influence on surface waters (Jasechko et al., 2016).

The new water fraction generates a storm response of individual events that is superimposed on the seasonal variation in base flows, with the highest peak flows tending to cluster in the wettest years (e.g., 2011 and 2017). Nevertheless, the evolution and operation of the network of runoff detention ponds had a non-stationary effect on storm runoff over the studied period, as more ponds have been added over time and their effectiveness and impact depend on how antecedent conditions and the sequencing of rainfall events fills the storage capacity. This highlights the importance of the memory effects of past management practices and the piecemeal, fragmented evolution of cumulative effects from contemporary management (e.g., Golden & Hoghooghi, 2018), information that should ideally be readily available but is often hidden in grey literature and archives.

This combination of separated urban storm drainage and groundwater inputs explains why the stream was usually relatively low in typical urban pollutants such as NO_3_^–^-N, P, and B due to the absence of continuous inputs of treated sewage following the closure of WWTPs in the catchment. The interaction between storm runoff and groundwater resulted in many solutes showing strong dilution with discharge increases. Despite the relatively low nutrient levels, the stream shows strong evidence of low oxygen concentrations in the summer, indicating both temperature effects and high ecosystem respiration. This is of particular concern, as the problem is likely to exacerbate with climate change, and the anticipated increased temperatures and lower summer flows have been recently shown to be causing global trends in increased stream anoxia (Zhi et al., 2023).

### Challenges to modeling

Traditional urban rainfall-runoff models remain useful tools for simulating the effects of urban storm drains and flood prediction (e.g., El Alfy, 2016; Ramly & Tahir, 2016). Usually, in such cases, a simplified representation of groundwater is not problematic as it represents only a minor component of flood flows. However, where predictions of low flows and their vulnerability to climate change are equally important to close the long-term water balance, alternative modeling tools are needed. One such alternative is using ensemble modeling approaches with either different models or different sets of calibrated parameters for the same model, providing a way to separately address these contrasting and increasingly important applied problems (e.g., Kobayashi et al., 2023; Nourani et al., 2021). Even so, if a more integrated understanding of urban hydrological systems is needed, then more sophisticated process-based modeling may be required, with a more explicit representation of drainage systems and groundwater processes. This is particularly the case when management interventions and land use change predictions are affecting both high flows and low flows.

In this study, our third specific research aim was to use exploratory modeling as a learning framework and to integrate our insights about the catchment’s response. In this regard, and despite its recent successful application in the Panke catchment in Berlin (Smith et al., 2023), we have seen that successfully calibrating HEC-HMS to the Wuhle proved to be difficult in a way that both storm flow and base flow components could be adequately captured. This likely reflects uncertainties over catchment size and any related interactions between the upper AQ2 aquifer that is hydraulically connected to the Wuhle stream network and the larger regional aquifer system (Limberg & Thierbach, 2002). Furthermore, potential groundwater abstraction via riverbank filtration from the Wuhle and the time-variant nature of the operation of storm water storage in detention ponds cannot be captured with the relatively simple modeling framework used here. These interactions may also have time-variant effects during drought periods. To capture such effects, a fully distributed process-based model of groundwater-surface water interactions may be needed such as PARFLOW or HydroGeoSphere (e.g., Bhaskar et al., 2015). However, such modeling would also need to be integrated with the capabilities of models such as SWMM (Rossman & Simon, 2022) and MIKE + (DHI, 2024) to parameterize the effects of non-stationarity in the management of the catchment, with increasing use of flood storage retention ponds in sustainable urban drainage, as well as local effects of groundwater pumping. This would also benefit from the storm drainage network being directly parameterized when such information is available (e.g., Ariano & Oswald, 2022).

A major challenge will be integrating algorithms for the different time dynamics of highly responsive urban streams (which respond to rainfall in minutes) and the slower (annual to decadal) changes in underlying aquifers. Indeed, the 12-h modeling step used in this study may have been a decision that compromised both high and flow simulation. Even with the best models derived in this study, the failure to simulate isotopes in stream flow indicates that process representation in the rainfall-runoff transformation is not adequately capturing the mixing of fluxes with catchment storage. This difference to the relative success of Smith et al. (2023) in modeling in the Panke is likely attributable to the fact that there around 90% of the flows are regulated and therefore any uncertainties over groundwater-surface water interactions had only minor effects on model results. This is not the case in the Wuhle catchment.

### Future needs

Globally, pressures on urban streams like the Wuhle will increase in the coming decades. The dual threats of climate change and urban expansion are likely to reduce the availability of water in urban areas. Despite this, societal demands increasingly highlight the importance of urban green/blue spaces for amenity, health, and biodiversity benefits in built-up environments (Braubach et al., 2017; Foster et al., 2011; Gunawardena et al., 2017). In the Berlin-Brandenburg area, there is compelling evidence that lower rainfall and higher evapotranspiration are causing streams to become increasingly intermittent (Kleine et al., 2021). This is because reduced groundwater recharge weakens groundwater-surface water connectivity and increases the frequency and longevity of low flow periods (Smith et al., 2021). Maintaining urban river corridors as important green/blue spaces for esthetic, recreational, and biodiversity goals is likely to require management changes that reduce water abstractions and/or increase regulation from water imports (Foster et al., 2011; Oswald et al., 2023). Historically, in Berlin, this has been achieved by using treated wastewater effluent; the neighboring Panke and Erpe catchments are both mostly sustained by wastewater (Kuhlemann et al., 2022). However, while this wastewater supplements flow, it can also lead to the deterioration of water quality in a way that impacts the in-stream ecology (Warter et al., 2024). Additional benefits may come from the ongoing disconnection of existing urban drainage schemes in Berlin as older urban areas are re-developed and the use of sustainable urban drainage increases recharge.

As with other cities, future management will require a fuller knowledge of urban water courses and how regulated flows interact with hydrological flow paths in urban green spaces and the rural parts of peri-urban catchments. This will require better data and better models that can integrate engineered components and more natural areas. Such information will be fundamental to provide an evidence base for improved management. In this regard, recent intensification in scientific monitoring efforts through the investigation of urban critical zones is to be welcomed (Arora et al., 2023; Ring et al., 2023). However, regardless of a better understanding, climate change makes the threat of greater intermittent flows more likely in Berlin. It is thus likely that hard choices will have to be made between either maintaining urban streams more perennial through flow regulation or allowing them to dry out more frequently in summer, with implications for urban riparian environments.

## Conclusions

In this work, we have explored the complex hydrological dynamics of the Wuhle stream and its catchment in the Berlin-Brandenburg area through integrated synthesis, analysis of data, and semi-distributed process-based rainfall-runoff modeling. This was achieved via the integration of multiple datasets, including pre-existing data produced by public offices (e.g., landscape characteristics, hydrometric, and meteorological data), and new water quality and stable isotope data. The interpretation of this data was aided by output from statistical analysis (PCA), estimation of the Young Water Fraction (YWF) and the Mean Transit Time (MTT), a filter-based hydrograph separation (HydRun), and a semi-distributed, process-based rainfall-runoff model (HEC-HMS). The results highlighted the complexity of urban hydrology in large evolving cities, where the subtle interactions between natural and engineered systems may lead to hydrological responses that are hard to disentangle given the heterogeneity of urban landscapes.

Looking ahead, mitigation of growing pressures on urban streams warrants a re-evaluation of water management strategies. These will play a critical role, especially in the context of urban expansion and climate change, under which altered precipitation, infiltration, and evapotranspiration patterns are to be expected. One reason for this is that fast-draining urban catchments suffer from reduced groundwater recharge below sealed surfaces, which is especially exacerbated by drought periods. Because groundwater processes are crucial for streams like the Wuhle, the increasing intermittency of such streams raises concerns about the sustainability of urban river corridors and underscores the importance of informed decision-making in managing water resources.

The complex hydrological response of this catchment and the limited readily available data on the cumulative effects of the evolution of water management, together with the modest success of a traditional, semi-distributed, process-based rainfall-runoff model (HEC-HMS), highlighted the need for more advanced data-driven models. These should be ideally equipped to deal with non-stationarity, with a sophisticated representation of urban groundwater to simultaneously address the impacts of management interventions and land use changes on both high and low flows. To successfully calibrate and validate such models, continuous and sustained monitoring of urban streams will remain essential and help to achieve the goal of maintaining the ecological balance while at the same time satisfying basic human needs and providing a range of ecosystem services.

### Supplementary Information

Below is the link to the electronic supplementary material.

Supplementary file1 (DOCX 6220 KB)

## Data Availability

Stable isotope and water physio-chemistry data have been produced by our research group and can be provided upon request.
