# Supplementary material for: Integrated monitoring and modeling to disentangle the complex spatio-temporal dynamics of urbanized streams under drought stress
Source: Environ Monit Assess. 2024 May 20;196(6):560. doi: 10.1007/s10661-024-12666-3 (PMC11106154; doi:10.1007/s10661-024-12666-3)
Supplement: Supplementary file 1 — Supplementary file1 (DOCX 6220 KB) [file 10661_2024_12666_MOESM1_ESM.docx]

SUPPLEMENTARY INFORMATION

**Integrated monitoring and modeling to disentangle the complex spatio-temporal dynamics of urbanized streams under drought stress**

Gregorio Alejandro López Moreira Mazacotte^1*^, Doerthe Tetzlaff^1,2^, Christian Marx^3^, Maria Magdalena Warter^1^, Songjun Wu^1,2^, Aaron Andrew Smith^1^, Chris Soulsby^1,3,4^

^1^ Department of Ecohydrology and Biogeochemistry, Leibniz Institute of Freshwater Ecology and Inland Fisheries (IGB), Berlin, Germany;

^2^ Geography Department and IRI THESys, Humboldt-Universität zu Berlin, Berlin, Germany;

^3^ MOSAIC research group - Modelling surface and groundwater with isotopes in urban catchments, Technische Universität Berlin, Berlin, Germany;

^4^ Northern Rivers Institute, School of Geosciences, University of Aberdeen, Aberdeen, Scotland, United Kingdom

* Corresponding author: [gregorio.lopezmoreira@igb-berlin.de](mailto:gregorio.lopezmoreira@igb-berlin.de)

**Hydrologic Engineering Center - Hydrological Modeling System (HEC-HMS)**

The HEC-HMS was developed by the US Army Corps of Engineering for dendric watersheds, integrating various structural elements for comprehensive hydrological analysis—both short-term (event-based) and long-term (multi-year) (Feldman, 2000). HEC-HMS integrates structural elements for comprehensive hydrological analysis. It operates at a sub-catchment scale, managing water storage and fluxes. Reservoirs, diversions and point inflows can be included.

The model accounts for vegetation effects on water availability using a simple canopy bucket storage method, considering factors like maximum canopy storage, and root-water usage. Incoming precipitation fills the canopy storage until it reaches capacity. Excess precipitation is directed to the land surface, while evaporation from the canopy is determined by a reference evapotranspiration rate and a crop coefficient. In this respect, the model features methods to estimate evapotranspiration and storage (canopy and surface compartments), surface and subsurface flow (as direct flow), soil layer infiltration, groundwater percolation, base flow generation, and routing. These processes are parameterized for each sub-catchment. Outflow from sub-catchments is gathered by the stream network and directed downstream (Fig. S2).

Land surface is classified as either directly connected impervious surfaces or pervious surfaces. Precipitation on impervious surfaces translates to direct flow into the channel (Q_d_), whereas precipitation on pervious surfaces is managed by the model's hydrological partitioning structure and loss estimations. For simulations involving storage dynamics, fluxes, and tracer mixing, the model estimates hydrological loss using the Soil Moisture Accounting (SMA) method (Bennett and Peters, 2000).

Surface water on pervious surfaces are available for infiltration to the soil storage at the beginning of each time-step, with potential infiltration rates (*i_pot_(t)*) and capacity determined by the maximum infiltration rate (*i_max_*), and the current and maximum soil storage (*S(t)* and *S_max_*):

| $i_{pot}\left( t \right)=i_{max}\left( 1-\frac{S\left( t \right)}{S_{max}} \right)$ | (1) |
| --- | --- |

Water falling on pervious areas, that fails to immediately infiltrate, remains stored in surface depressions until it is either evaporated or infiltrates during subsequent time intervals. Excess surface water beyond the maximum depression storage is diverted to the channel as direct flow. The soil storage is divided into upper zone storage and tension zone storage. All water held within the soil storage contributes solely to evapotranspiration, while only the upper zone storage can percolate into deeper water reservoirs.

Flow into and out of deeper storages (two storages) is defined through the percolation rates:

| $P_{pot}\left( i,t \right)=P_{max}\left( i \right) \left( \frac{S\left( i,t \right)}{S_{max}\left( i \right)} \right)\left( 1-\frac{S\left( i+1,t \right)}{S_{max}\left( i+1 \right)} \right)$ | (2) |
| --- | --- |

where *P_pot_(i,t)* is the potential percolation from storage in layer *i*, and is dependent on the maximum possible percolation rate (*P_max_(i)*) and the ratio of current soil storage volumes (*S(i,t)*) to maximum storage volumes (*S_max_*) of layer *i* and layer *i+1*. The actual percolation (*P_act_(i,t)*) is the minimum of the potential percolation and the available water for percolation. The lateral flow (*Q_g_*) out of the deeper storages (not soil storage) is defined using the vertical water balance and a routing storage parameter (*GW_R_*):

| $Q_{g}\left( i,t+1 \right)=\frac{P_{act}\left( i-1,t \right)+S\left( i,t \right)-P_{pot}\left( i,t \right)-0.5Q_{g}\left( i,t \right)*t}{GW_{R}+0.5*t}$ | (3) |
| --- | --- |

with the total volume (*V*) of water released from storage:

| $V\left( i,t \right)=0.5\left( GW_{Q}\left( i,t+1 \right)+GW_{Q}\left( i,t \right) \right)*t$ | (4) |
| --- | --- |

The transformation of sub-catchment hydrographs is done via the Snyder Unit Hydrograph method. This method utilizes unit hydrographs to convert runoff from the catchment into peak flows for sub-catchment hydrographs (Feldman, 2000). River flood routing through channelized sections is estimated using the Muskingum-Cunge approach (Cunge, 1969). To direct water flow, it relies on channel length, channel slope, Manning’s *n* coefficient, and channel shape. For ease of calculation, a rectangular channel shape was assumed, and automatic estimation of space and time step for routing was implemented to ensure numerical stability.

The Penman-Monteith method (Monteith, 1965) is used to estimate potential evapotranspiration using a combination of energy balance and mass transfer approaches (Allen et al., 1998). Required input includes short and longwave radiation, wind speed, air temperature, air pressure and vapor pressure in conjunction with a reference albedo and a reference crop coefficient.

**References**

Allen, R. G., Pereira, L. S., Raes, D., & Smith, M. (1998). Crop evapotranspiration —guidelines for computing crop water requirements. FAO Irrigation and Drainage Paper 56, 300 p. https://www.fao.org/3/X0490E/x0490e00.htm

Cunge, J. A. (1969). On the subject of a flood propagation computation method (Muskingum method). *Journal of Hydraulic Research*, *7*(2), 205–230. https://doi.org/10.1080/00221686909500264

205–230

Feldman, A. D. (2000, March). Hydrologic Modeling System HEC-HMS: Technical Reference Manual. U.S. Army Corps of Engineers. Retrieved 2024, February 6, from https://www.hec.usace.army.mil/software/hec-hms/documentation/HEC-HMS_Technical%20Reference%20Manual_(CPD-74B).pdf

Monteith, J. L. (1965). Evaporation and environment*. Symposia of the Society for Experimental Biology*, *19*, 205–234. https://repository.rothamsted.ac.uk/item/8v5v7

**Table S1. Catchment land use characteristics**

|  | **Wuhle (whole catchment)** | |
| --- | --- | --- |
|  | **Area (km^2^)** | **Area (%)** |
| **Urban** | 60.72 | 55.6% |
| **Non-Urban** |  |  |
| Annual crops | 27.87 | 25.5% |
| Pastures | 9.27 | 8.6% |
| Forests | 8.54 | 7.8% |
| Herbaceous vegetation | 1.20 | 1.1% |
| Water | 0.88 | 0.8% |
| Wetlands | 0.66 | 0.6% |
| **Subtotal Non-Urban** | 48.42 | 44.4% |
| **Total** | **109.14** | **100.0%** |

**Table S2. Calibrated parameters: Parameter calibration ranges**

| **Parameter No.** | **Process Method** | **Selected Method** | **Parameter** | **Range** |
| --- | --- | --- | --- | --- |
| 1 | Canopy | Simple Canopy | Initial Storage (%) | 60-80 |
| 2 | Canopy | Simple Canopy | Maximum Storage (mm) | 0.5-1.5 |
| 3 | Canopy | Simple Canopy | Crop Coefficient | 0.6-1.5 |
| 4 | Surface | Simple Surface | Initial Storage (%) | 60-80 |
| 5 | Surface | Simple Surface | Maximum Storage (mm) | 5-35 |
| 6 | Loss | Soil Moisture Accounting (SMA) | Initial Soil Content (%) | 60-80 |
| 7 | Loss | Soil Moisture Accounting (SMA) | Initial GW1 Content (%) | 60-80 |
| 8 | Loss | Soil Moisture Accounting (SMA) | Initial GW2 Content (%) | 60-80 |
| 9 | Loss | Soil Moisture Accounting (SMA) | Maximum Infiltration Rate (mm/h) | 120-150 |
| 10 | Loss | Soil Moisture Accounting (SMA) | Soil Storage Capacity (x) (mm) | 80-180 |
| 11 | Loss | Soil Moisture Accounting (SMA) | Tension Storage Capacity (mm) | 0-x |
| 12 | Loss | Soil Moisture Accounting (SMA) | Soil Percolation Rate (mm/h) | 5-30 |
| 13 | Loss | Soil Moisture Accounting (SMA) | GW1 Storage Capacity (mm) | 0-500 |
| 14 | Loss | Soil Moisture Accounting (SMA) | GW1 Storage Coefficient (h) | 200-1500 |
| 15 | Loss | Soil Moisture Accounting (SMA) | GW1 Percolation Rate (mm/h) | 0-25 |
| 16 | Loss | Soil Moisture Accounting (SMA) | GW2 Storage Capacity (mm) | 0-500 |
| 17 | Loss | Soil Moisture Accounting (SMA) | GW2 Storage Coefficient (h) | 3000-7000 |
| 18 | Loss | Soil Moisture Accounting (SMA) | GW2 Percolation Rate (mm/h) | 0-5 |
| 19 | Transform | Snyder Unit Hydrograph | Slope (m/km) | 1.5-3.5 |
| 20 | Base flow | Linear Reservoir | GW1 Initial Discharge (m^3^/s) | 0-1 |
| 21 | Base flow | Linear Reservoir | GW2 Initial Discharge (m^3^/s) | 0-1 |
| 22 | Base flow | Linear Reservoir | GW3 Initial Discharge (m^3^/s) | 0-1 |
| 23 | Base flow | Linear Reservoir | GW3 Base Flow Fraction | 0-1 |

**
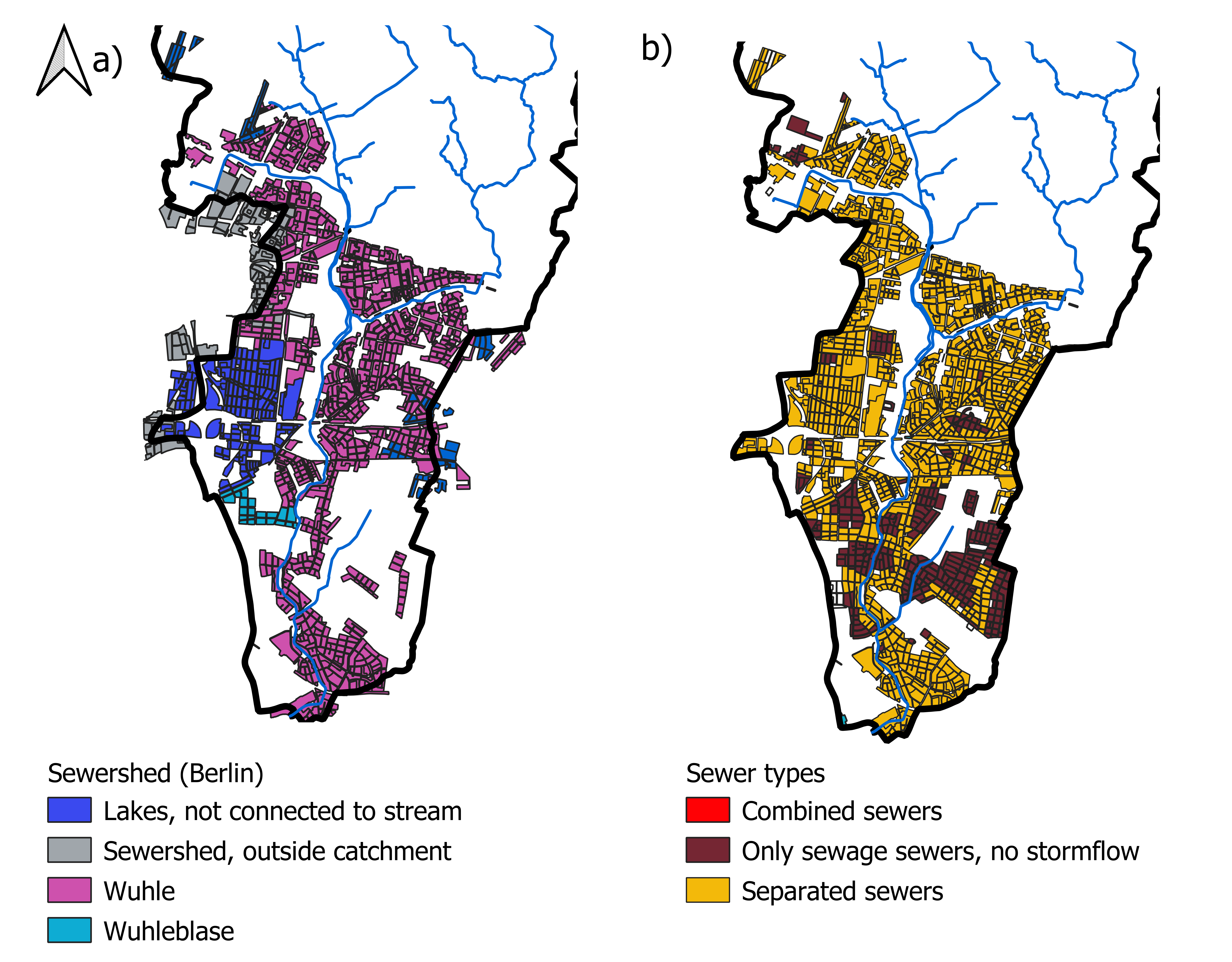
Fig. S1** Maps of a) the sewersheds; and b) the sewer system types in the lower Wuhle catchment (within the city limits of Berlin)

**
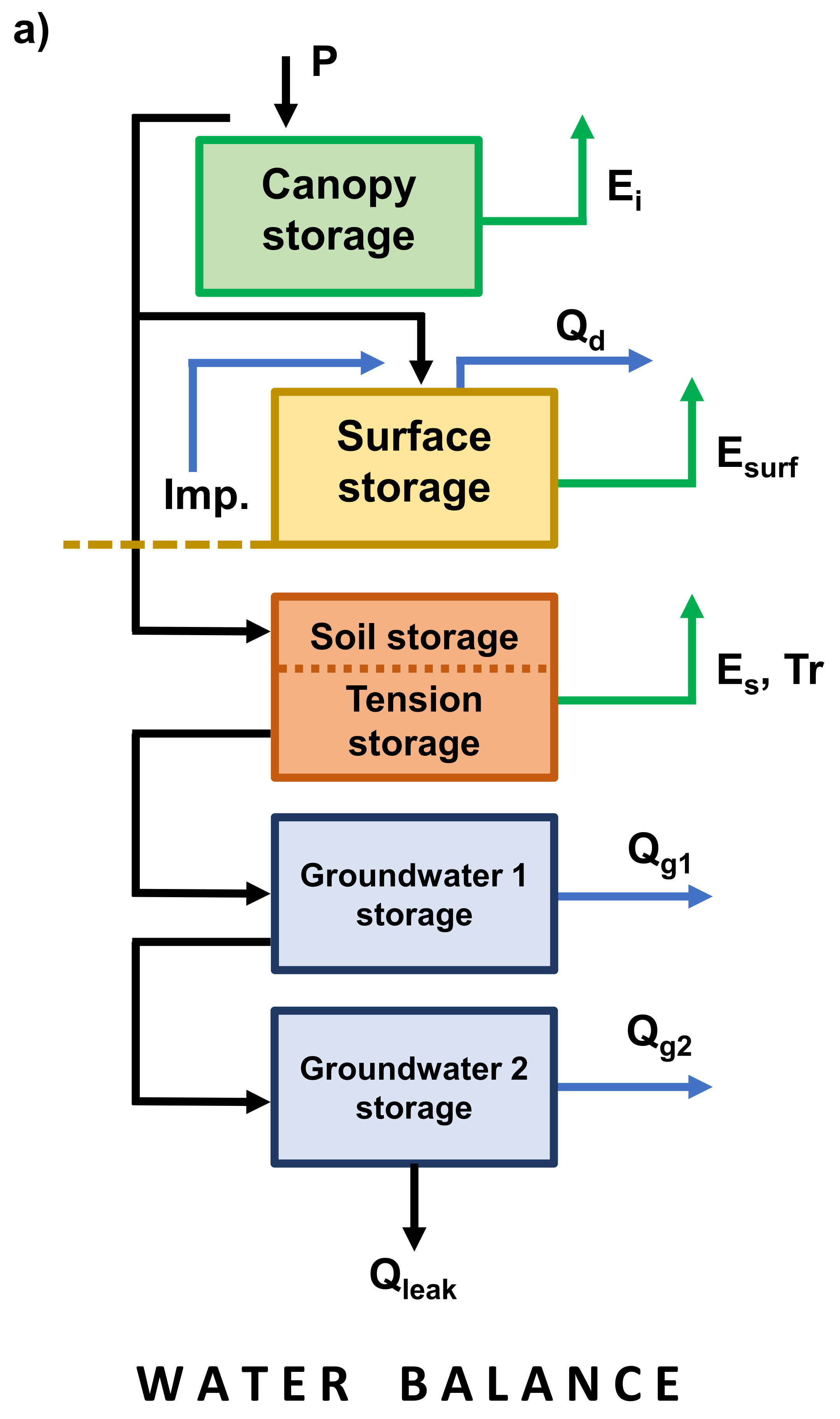

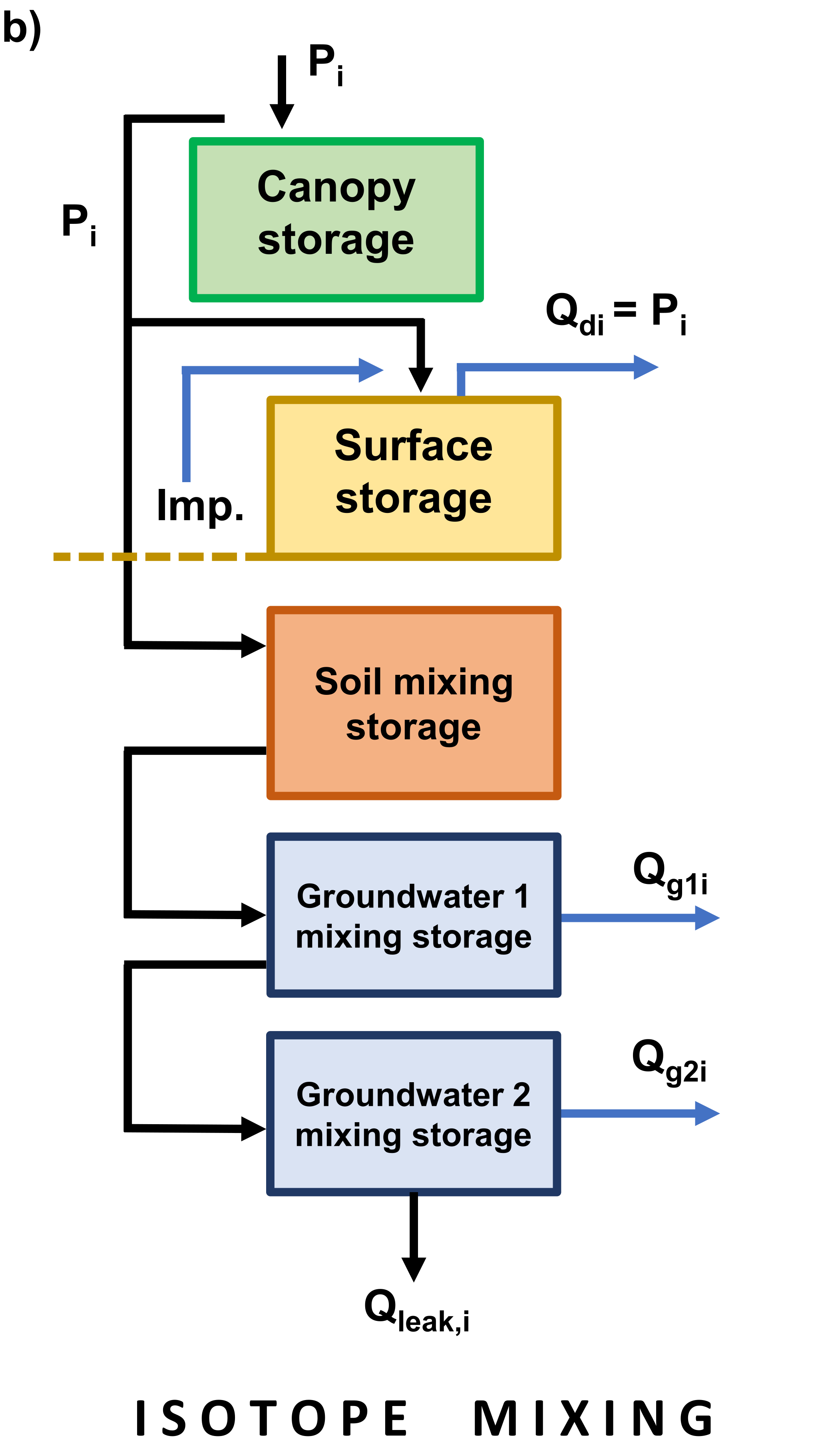
**

**Fig. S2** Model structure diagram of (a) HEC-HMS semi-distributed rainfall-runoff process-based model, and (b) isotope mixing model

**

**

**Fig. S3** Measured discharge, and measured vs modeled oxygen-18 signals from HEC-HMS partitioning
